# Two new approaches for the visualisation of models for network meta-analysis

**DOI:** 10.1186/s12874-019-0689-9

**Published:** 2019-03-18

**Authors:** Martin Law, Navid Alam, Areti Angeliki Veroniki, Yi Yu, Dan Jackson

**Affiliations:** 1 MRC Biostatistics Unit, Cambridge, UK; 2 Statistical Laboratory, University of Cambridge, Cambridge, UK; 3 Li Ka Shing Knowledge Institute, St. Michael’s Hospital, Toronto, Canada; 4 Department of Primary Education, School of Education, University of Ioannina, Ioannina, Greece; 5 School of Mathematics, University of Bristol, Bristol, UK; 6 Statistical Innovation Group, Advanced Analytics Centre, AstraZeneca Cambridge, Cambridge, UK

**Keywords:** Network meta-analysis, Network analysis, Visualisation

## Abstract

**Background:**

Meta-analysis is a useful tool for combining evidence from multiple studies to estimate a pooled treatment effect. An extension of meta-analysis, network meta-analysis, is becoming more commonly used as a way to simultaneously compare multiple treatments in a single analysis. Despite the variety of approaches available for presenting fitted models, ascertaining an intuitive understanding of these models is often difficult. This is especially challenging in large networks with many different treatments. Here we propose two visualisation methods, so that network meta-analysis models can be more easily interpreted.

**Methods:**

Our methods can be used irrespective of the statistical model or the estimation method used and are grounded in network analysis. We define three types of distance measures between the treatments that contribute to the network. These three distance measures are based on 1) the estimated treatment effects, 2) their standard errors and 3) the corresponding p-values. Then, by using a suitable threshold, we categorise some treatment pairs as being “close” (short distances). Treatments that are close are regarded as “connected” in the network analysis theory. Finally, we group the treatments into communities using standard methods for network analysis. We are then able to identify which parts of the network are estimated to have similar (or different) treatment efficacy and which parts of the network are better identified. We also propose a second method using parametric bootstrapping, where a heat map is used in the visualisation. We use the software R and provide the code used.

**Results:**

We illustrate our new methods using a challenging dataset containing 22 treatments, and a previously fitted model for this data. Two communities of treatments that appear to have similar efficacy are identified. Furthermore using our methods we can identify parts of the network that are better (and less well) identified.

**Conclusions:**

Our new visualisation approaches may be used by network meta-analysts to gain an intuitive understanding of the implications of their fitted models. Our visualisation methods may be used informally, to identify the most salient features of the fitted models that can then be reported, or more formally by presenting the new visualisation devices within published reports.

**Electronic supplementary material:**

The online version of this article (10.1186/s12874-019-0689-9) contains supplementary material, which is available to authorized users.

## Background

Meta-analysis is a popular technique for combining the results from multiple two-arm studies that compare a single pair of treatments. Here each included study provides an estimated treatment effect and its associated precision. Standard methods for meta-analysis result in a weighted average of these study specific estimated treatment effects.

The concept of aggregating multiple two-arm studies has been extended to networks of evidence that simultaneously compare multiple (more than two) treatments. This may include multi-arm studies that examine more than two treatments. This extension is called network meta-analysis [1, 2], where estimates of the relative treatment effect of all possible pairs of treatments are simultaneously obtained. This includes pairs of treatments that have not been compared directly in any trial. However, it can be difficult to interpret fitted models for network meta-analysis, especially when many treatments are included. For example, in large networks it is typically hard to determine which treatments are estimated to have similar efficacy, which parts of the network are well identified, and so on. The aim of this paper is to provide two new visualisation methods to help both analysts and the consumers of network meta-analyses better understand the implications of their fitted model.

A wide variety of models and estimation methods for network meta-analysis are available. However, all we assume is that we either have, or can deduce, the estimated relative treatment effects (and the corresponding standard errors) for all possible treatment comparisons in the network. We will show how these quantities can be calculated from standard network meta-analysis output and these quantities should, in any case, be reported when presenting the results from network meta-analyses. Hence for our purposes it does not matter what statistical methodology was used. All we require is that a network meta-analysis model has been fitted.

**Fig. 1:**
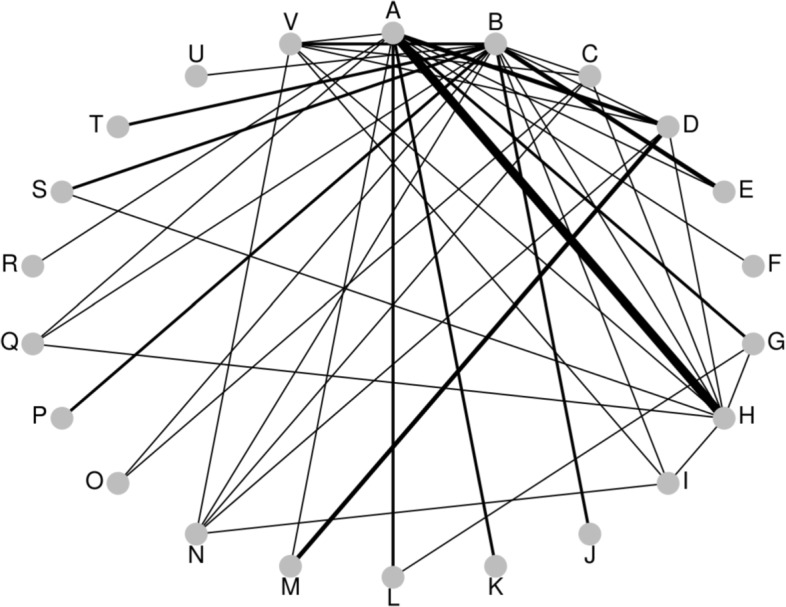
Treatment comparisons: Osteoarthritis of the knee. Network diagram for example dataset of treatments for osteoarthritis of the knee. Edge width represents number of direct comparisons. A: standard care; B: placebo; C: no medication; D: acupuncture; Ebalneotherapy; F: braces; G: aerobic exercise; H: muscle exercise; I: heat treatment; J: insoles; K: tai chi; L: weight loss; M: sham acupuncture; N: ice/cooling; O: interferential; P: laser; Q: manual; R: neuromuscular electrical stimulation (NMES); S: pulsed electrical stimulation (PES); T: PEMF; U: static magnets; V: transcutaneous electrical nerve stimulation (TENS)

Our intention is to visualise fitted network meta-analysis models, rather than the data that was used to fit them. Visual displays of the structure of the data are also important and ‘network diagrams’ are often provided. A variety of software is available for producing these diagrams [3–5] and in Fig. 1 we show a network diagram, created using the R [6] package *pcnetmeta* [4], for the dataset that we will later use to illustrate our methods. Here, each edge represents the presence of a direct comparison, and the thickness is proportional to the number of direct comparisons. A number of different conventions are possible when using network diagrams, such as providing the number of direct comparisons on the edges or displaying multi-arm studies using polygons. See Chaimani et al. [5] for a discussion of the possible conventions that can be used when presenting network diagrams and also a variety of other types of graphical displays. Network diagrams convey the main characteristics of the data, rather than results from statistical analyses that provide our focus here.

**Fig. 2:**
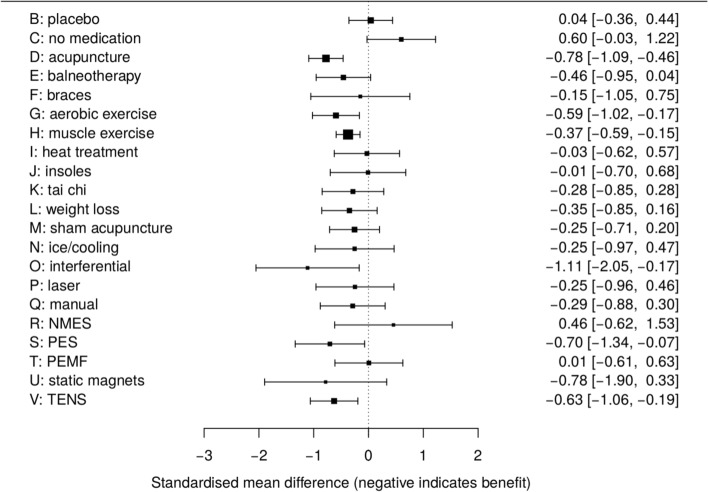
Forest plot of results compared to reference treatment A, standard care. NMES, neuromuscular electrical stimulation; PES, pulsed electrical stimulation; PEMF, pulsed electro- magnetic fields; TENS, transcutaneous electrical nerve stimulation

It appears that there is currently no standard approach to visually displaying the results from network meta-analyses. A variety of contrasting possibilities have therefore been used in practice. For example forest plots, ubiquitous in pairwise meta-analysis, can be repurposed in network meta-analysis to visually compare a reference treatment to all the others. Figure 2 is an example of such a forest plot, where we show the estimated treatment effects and 95% confidence intervals for all treatments, relative to standard care, for the fitted model that we will use illustrate our methods below. It is possible to extend this idea and include all relative effect estimates in a single forest plot (for example Wang et al. [7], their Figure 2; Wu et al. [8], their Figure 4; Tricco et al. [9], their Figure 2). Forest plots such as these are useful but it is not easy to then visualise the implications of the fitted model for the network as a whole. Wu et al. [8] also use the additional visual device of showing network diagrams that have estimated treatment effects and confidence intervals displayed on the network edges (their Figure 2). However this results in network diagrams that display a very large amount of information that is difficult to visualise. Wu et al. [8] also plot Bayesian ranking probabilities (their Figure 3). In a Bayesian framework, rankings can also be produced using the surface under the cumulative ranking curve (SUCRA) [10]. Figure 3 of Dulai et al. [11] is an interesting example of a visual display of SUCRA rankings, where a scatterplot of SUCRA rankings for safety are plotted against SUCRA rankings for efficacy. While plots of ranking probabilities may be useful to gain an understanding of which treatments are likely to be the most effective it remains, at best, difficult to visualise which treatments are most similar in terms of their estimated efficacy, the extent to which each pairwise comparison is well identified, and so on.

Another way to communicate the results from network meta-analyses is to present the results numerically in tables. Estimates can be tabulated with respect to a single reference treatment only (Wu et al. [8], their Table 2) or for all pairwise comparisons (Wang et al. [7] their Table 1; Tricco et al. [9] their Table 2). Tables of network meta-analysis results may also be presented by allocating one row and column to each treatment, and showing the inferences for each comparison in the appropriate table entry (Dulai et al. [11] their Figure 2; Stegeman et al. [12], their Table 3). However visualising the implications of tables of results when there are many treatments in the network, may be a daunting task.

In short, although a variety of ways to display fitted models for network meta-analysis have been proposed, none of these ideas provide simple or intuitive approaches for visualising these models. This paper exploits ideas from network analysis to develop two new methods that display communities of treatments that are identified as being similar to each other, using three distance measures. We will therefore be able to easy identify which treatments are estimated to have similar efficacy, which parts of the network are well identified, and so on.

A recently developed method is that of Rücker et al. [13], which separates treatments into a hierarchy of efficacy. This is in contrast to our methods, which seek to group similar treatments. Although the method proposed by Rücker *et al* uses adjacency matrices based on treatment efficacy, as we propose, and so is in some respects similar to our methods, we prefer our approach as it uses more sophisticated community detection algorithms and visualises several different aspects of the fitted model.

The rest of the paper is set out as follows. We introduce the concepts necessary to understand our approach to visualisation using community detection — for example, distance measures, distance matrices and adjacency matrices. We then describe community detection itself and explain the approach we have used to group the treatments into communities, modularity maximisation. We describe our two visualisation methods and apply them to a challenging example network that has been analysed previously. We conclude with a discussion.

## Methods

We will use an artificial example to explain our methods. This example involves only four treatments and is sufficiently simple that we do not require methods such as ours to visualise it; we present it for didactic purposes only.

Without loss of generality, we take treatment *A* as the reference treatment. Models for network meta-analysis can then be described using treatment effects of the other treatments relative to this reference treatment (treatments *B*, *C*, *D*, and so on). These treatment effects are usually denoted as *δ*^*AB*^,*δ*^*AC*^,*δ*^*AD*^ and referred to as basic parameters [14*–*17]. The primary inferences are made by estimating these basic parameters and their covariance matrix, which immediately results in inferences for all treatment effects relative to *A*. The inferences for the other treatment effects are made using appropriate linear combinations of these basic parameters, for example the treatment effect of *C* relative to *B* is *δ*^*AC*^−*δ*^*AB*^. For our artificial example, suppose that such a model for network meta-analysis containing four treatments has been fitted, where we estimate ***δ***=(*δ*^*AB*^,*δ*^*AC*^,*δ*^*AD*^)^*T*^ as

1 $$  \hat{\boldsymbol{\delta}} = \left(\begin{array}{c} 0.30\\ 0.50\\ 0.45 \end{array} \right){;} \text{Var}\left(\hat{\boldsymbol{\delta}}\right)=\hat{\boldsymbol{V}}= \left(\begin{array}{c c c } 0.041 & 0.019 & 0.005\\ 0.019& 0.102& 0.004\\ 0.005& 0.004& 0.026 \end{array} \right).  $$

For our purposes it does not matter what type of model or estimation method was used result in (1). To visualise the implications of this model, we define three distance measures. It is possible to use other measures of distance when using our methods and we return to this issue in the discussion and the Additional file 1. Each of these distances measures result in a *t*×*t* distance matrix that we call a ***D*** matrix, where *t* is the number of treatments included in the network. The entries of these ***D*** matrices, denoted *d*_*ij*_, will then be the distance between the *i*th and *j*th treatments. For example, *d*_24_ will denote the distance between the second and fourth treatments, i.e. treatments *B* and *D*. We will define *d*_*ii*_=0 for *i*=1,2,⋯*t*, so that the distance from any treatment to itself is zero. We will also ensure that *d*_*ij*_=*d*_*ji*_ for *i*≠*j*, hence we use distance measures that are symmetrical.

### Three measures of distance between treatments in the network

The most obvious measure of the difference between two treatments in the fitted model is the absolute value of their estimated relative treatment effect. This distance is used in order to visualise which treatments are estimated to have similar efficacy. From (1), this measure of distance results in the ***D*** matrix

2 $$ \boldsymbol{D}_{1} = \left(\begin{array}{cccc} 0 & 0.30 & 0.50 & 0.45 \\ 0.30 & 0 &0.20 &0.15 \\ 0.50 & 0.20 & 0 & 0.05 \\ 0.45 & 0.15 & 0.05 & 0 \end{array} \right).  $$

The entries in the first row and column of ***D***_1_ in (2) are simply the absolute values of the estimated basic parameters given in (1). The other entries of ***D***_1_ are the absolute values of the appropriate linear combinations of estimated basic parameters that provide the appropriate estimated treatment effect. For example $d_{23} = d_{32} = | \hat {\delta }^{AC} - \hat {\delta }^{AB} | = |0.5-0.3|=0.20$. These distances are our first way of measuring distance in the network. Small distances indicate that treatments are “close" (similar estimated treatment efficacy). Small distances of the two other measures of distance that follow also indicate that treatments are close. An example of how to obtain the entries of a generic **D**_1_ matrix is provided in the Additional file 1.

Another way to measure the distance between treatments is the standard error of the estimated treatment effects. This distance is used in order to visualise which parts of the network are more accurately identified. From (1), this measure of distance results in the ***D*** matrix

3 $$  \boldsymbol{D}_{2} = \left(\begin{array}{cccc} 0 & 0.202 & 0.319& 0.161\\ 0.202 & 0 & 0.324 & 0.239\\ 0.319 & 0.324 &0 &0.346\\ 0.161 &0.239 &0.346 &0 \end{array} \right),\ \text{to 3 d.p.}  $$

The entries in the first row and column of ***D***_2_ in (3) are simply the square-root of the diagonal entries of $\hat {\boldsymbol {V}}$ in (1). The other entries of ***D***_2_ are the standard errors of the appropriate linear combinations of estimated basic parameters that provide the appropriate treatment effect. For example

$$\begin{array}{*{20}l} d_{23} & =d_{32} =\sqrt{\text{Var}\left(\hat{\delta}^{\text{AC}}- \hat{\delta}^{\text{AB}}\right)}=\sqrt{\text{Var}\left(\hat{\delta}^{\text{AB}}- \hat{\delta}^{\text{AC}}\right)} \\ & = \sqrt{\text{Var}\left(\hat{\delta}^{\text{AC}}\right) + \text{Var}\left(\hat{\delta}^{\text{AB}}\right) \!- 2\times \text{Cov}\left(\hat{\delta}^{\text{AC}}, \hat{\delta}^{\text{AB}}\right)} \\ & =\sqrt{0.041+0.102-2\times0.019} = 0.324 \text{, to 3 d.p.} \end{array} $$

These distances (the standard errors of the estimated treatment effects) are our second way of measuring distance in the network. Small distances indicate that treatments are close, where this closeness here is taken to indicate that their relative treatment effect is well identified. Treatments may be close according to one distance measure — for example, by standard error, meaning that pairwise treatment effect is well-identified — and not close at all by another — for example, by treatment effect, meaning that the estimated difference in treatment effect is great. Any distance measurement may be used, given that its choice can be justified, and more examples are given in the discussion.

Our third and final measure of distance is based upon the two-tailed p-values that are calculated using the absolute estimated treatment effects and their standard errors using the first two distances. This distance is used in order to visualise which treatments are estimated to have similar efficacy in terms of their statistical significance, rather than their estimated treatment effect. Although we would align ourselves with those who emphasise estimation over testing, decision making is often based on the results of hypothesis tests and so we include this distance for the benefit of those who make decisions using this type of criterion. Further, the reader should be aware of the fact that, under the null hypothesis and when using a continuous test statistic, *p* values follow a uniform distribution on [0, 1]; this result, combined with issues relating to repeated testing, may serve to discourage analysts from drawing strong conclusions from the occasional small (or large) *p* value. The ratio of the entries of the ***D*** matrices in (2) and (3) provide the usual test statistics *Z*_*ij*_, for *i*≠*j*, and the corresponding two-tailed p-values are *P*_*ij*_=2*Φ*(−*Z*_*ij*_), where *Φ*(·) is the standard normal cumulative distribution function. These p-values are not immediately appropriate measures of the distances between treatments, because a smaller p-value means that there is stronger statistical significance, which means that the treatments are more different and so further apart. This is in contrast to the previous two distance measures, where small values indicate similarity. We therefore use the complement of these p-values, *d*_*ij*_=1−*P*_*ij*_=1−2*Φ*(−*Z*_*ij*_), for *i*≠*j*, where we further define *d*_*ii*_=0. For our artificial example the resulting distance matrix is

4 $$  \boldsymbol{D}_{3} = \left(\begin{array}{cccc} 0 & 0.862 & 0.882 &0.995\\ 0.862 & 0 & 0.463 & 0.470\\ 0.883 & 0.463 & 0 & 0.115\\ 0.995& 0.470 & 0.115 & 0\\ \end{array} \right),\ \text{to 3 d.p.}  $$

When explaining how to derive our three distances, we have assumed that the fitted network meta-analysis model is parameterised using basic parameters. This is typically the case but need not be so, for example if an arm-based analysis [18] has been performed. All that we require is that distance matrices using our three measures can be calculated from the fitted model. The three types of distance matrices contain very straightforward quantities and it should be equally easy to calculate them when using any network meta-analysis methodology. The code we provide in the Additional file 1 takes (1) as the input and obtains the three distance matrices from these.

### Forming adjacency matrices

Our three measures of distance are not immediately useful in the network analysis methods for forming communities that follow. This is because these methods are intended to be applied to networks where some, but not all, vertices (in our context, treatments) are connected. Distance matrices (2), (3) and (4) indicate that all treatments are connected, though some are further away from each other than others. In order to create networks where not all vertices are connected, we will use a threshold to determine whether or not treatments are close enough together to be considered connected. We use a variety of thresholds and all three types of distance measures. However for didactic purposes, let us consider using just the first distance matrix ***D***_1_ (2) and assume that estimated relative treatment effects that are less than 0.4 are considered close enough to be connected, but those greater than 0.4 are not. For example, readers might like to imagine that in our artificial example, the estimated effects are standardised mean differences, and treatment effects on this scale that are less than 0.4 are often considered moderate. We therefore form an ‘adjacency matrix’ from (2), where all off-diagonal entries of ***D***_1_ that are less than or equal to the threshold give rise to entries of value one in the corresponding adjacency matrix; all other entries are set to zero. This results in the adjacency matrix **A**:

5 $$  \boldsymbol{A} = \left(\begin{array}{cccc} 0 & 1 &0 &0 \\ 1 & 0 &1 &1 \\ 0 & 1 & 0 & 1 \\ 0 & 1 & 1 & 0 \end{array} \right).  $$

**Fig. 3:**
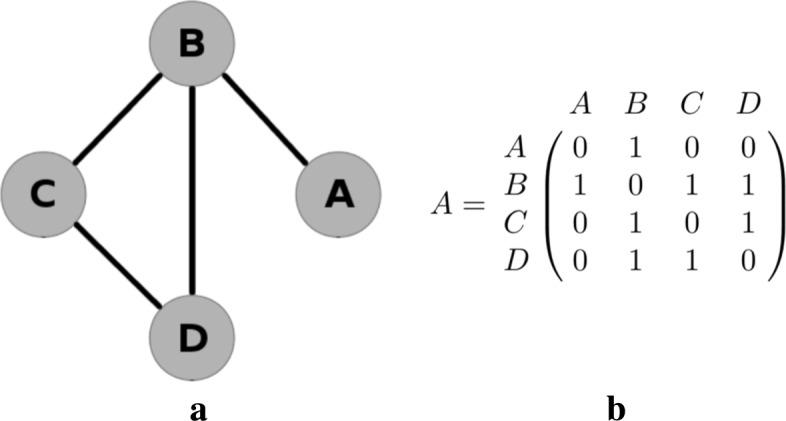
Network diagram and corresponding adjacency matrix for a basic network

Matrix ***A*** is symmetric, which is the case for all adjacency matrices used in our methodology because our three measures distances are symmetric. In general however, adjacency matrices need not be symmetric in network theory. The network corresponding to the adjacency matrix (5) is shown in Fig. 3.

### Network analysis and community detection

**Fig. 4:**
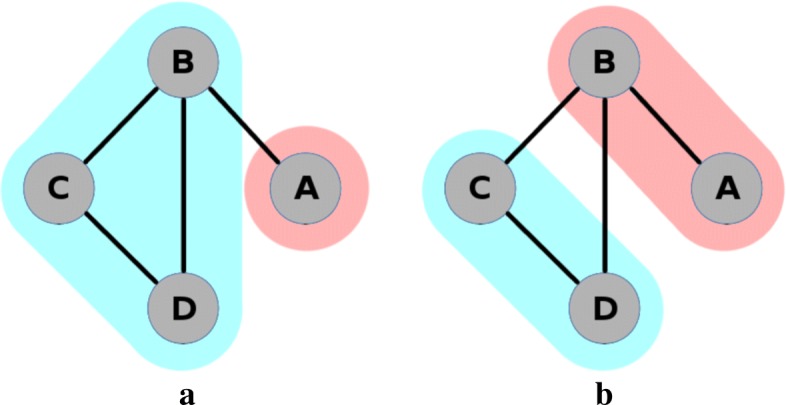
Two possible community structures for example network

In network analysis, vertices (or “nodes”) are connected by edges. The connections between the vertices in the network are described using adjacency matrices. We will use the notation *v*_*i*_ to indicate the *i*th vertex where, in the context of network meta-analysis, vertices represent treatments. For example, *v*_1_ will represent treatment A, *v*_2_ will represent treatment B, and so on. In network analysis, a community is a group of vertices that are placed together based on the properties of the edges within that network. Figure 4 shows the same network as in Fig. 3, with two possible community structures superimposed. Each vertex is placed in exactly one community. The first community structure (Fig. 4a) places treatment A in a community by itself, and the other three treatments in a second community. The second community structure (Fig. 4b) places treatments A and B in the first community and treatments C and D in the second. Other community structures are also possible, including the extremities of placing all four treatments in separate communities, or all treatments in a single community. We use the notation *C*_*i*_ to denote the community that contains vertex *v*_*i*_. For example, for the first community structure described above (Fig. 4a), we have *C*_1_=1 and *C*_2_=*C*_3_=*C*_4_=2, and in Fig. 4b we have *C*_1_=*C*_2_=1 and *C*_3_=*C*_4_=2. Network analysis provides us with methods to determine if the first of these community structures is considered a better description of the connection density than the second, or indeed if any other community structure describes this even better.

A common method of community detection is to calculate the *modularity* of each possible community structure. A community structure with high modularity has a high density of edges within communities, and few edges between communities. The community structure of interest is one that maximises the modularity. There may be more than one community structure that maximises modularity. Other community detection methods exist [19], but modularity maximisation remains widely used. We will adopt this well known approach here, and explain it in more detail below.

### Modularity

For simplicity, we describe what is meant by modularity for unweighted adjacency matrices, that is, for networks where vertices are regarded as either connected or unconnected. We define the total number of edges in the network to be *m*; for the network in Fig. 3 we have *m*=4. We define the number of edges connected to vertex *v*_*i*_ (its *degree*) to be *k*_*i*_; for the network in Fig. 3 we have *k*_1_=1,*k*_2_=3,*k*_3_=2 and *k*_4_=2. For a given network and community structure, the number of edges within communities is

6 $$  O=\frac{1}{2}\sum\limits_{i=1}^{t} \sum\limits_{j=1}^{t} A_{ij}\delta\left(C_{i}, C_{j}\right),  $$

where *δ*(*a,b*) is the Kronecker delta. The purpose of the half in (6) is to account for double counting, because in the double summation we count both ends of edges separately, and so we count each edge twice.

Assuming that all edges are equally likely to end at any vertex, the probability that one particular edge leads to vertex *v*_*j*_ is *k*_*j*_/2*m*. Vertex *v*_*i*_ has *k*_*i*_ edges, and so the expected number of edges from vertex *v*_*i*_ to *v*_*j*_ is *k*_*i*_*k*_*j*_/2*m*. The total expected number of edges connecting vertices from the same community is therefore

7 $$\begin{array}{*{20}l}  E=\frac{1}{2}\sum\limits_{i=1}^{t} \sum\limits_{j=1}^{t} \frac{k_{i} k_{j}}{2m} \delta(C_{i}, C_{j}) \end{array} $$

As in Eq. (6), the purpose of the half in (7) is to account for double counting.

The modularity is obtained by subtracting (7) from (6) and dividing by the total number of edges *m*, to give (*O*−*E*)/*m* or

8 $$  Q=\frac{1}{2m}\sum\limits_{i=1}^{t} \sum\limits_{j=1}^{t} \left(A_{ij} - \frac{k_{i} k_{j}}{2m} \right) \delta(C_{i}, C_{j}).  $$

The modularity *Q* is therefore essentially an ‘ *O*−*E* statistic’, a type of statistic widely used in statistics. By dividing by *m*, the modularity is the observed proportion of edges within communities minus the corresponding expected proportion of edges. A community structure that describes the density of the edges in the network well has a high modularity relative to that of others.

The optimal community structure is defined as the one that provides the maximum possible modularity for a given network. In relatively small networks, this optimal structure can be found by calculating the modularity for every possible community and taking the community structure with the largest value. Since *m* is is fixed for any given network, maximising the modularity is equivalent to maximising the ‘ *O*−*E* statistic’. In other applications where networks may contain hundreds, or even thousands, of vertices, this exhaustive approach will not feasible. It is then possible to use a heuristic algorithm to search through a smaller subset of possibilities [19]. In our code we allow the use of one such heuristic algorithm, because it could be useful in applications where there are very many treatments and many possible community structures. However it will only be in extreme instances where an algorithm will be necessary for computational reasons in network meta-analysis applications. The modularity of each of the community structures in Fig. 4 is *Q*_1_=−0.03125 and *Q*_2_=0. Thus the second community structure has a slightly greater modularity than the first, and so we would take this grouping to be the better representation of the network. Details of this calculation are shown in the Additional file 1.

### The first visualisation method

The first of our two visualisation methods simultaneously displays three separate community structures based on the three distance measures described above. This is because our three distances describe different aspects of the fitted model and we suggest that it is desirable to examine all of them. As explained above, when using algorithms for community detection it is necessary to remove some connections to avoid having every vertex directly connected. For this purpose we suggest using a threshold to dichotomise the measures into “close" (connected) and “not close" (unconnected). We use thresholds that are particular quantiles of the empirical distances (as contained in distance matrices for our artificial example in equations 2, 3 and 4). These matrices are symmetrical and so when determining an appropriate quantile we use just the upper triangle part of ***D*** matrices, excluding the main diagonal.

Once an appropriate threshold has been computed for each distance measure, adjacency matrices can be obtained and community detection algorithms applied. We have found it useful to explore the use of a variety of quantiles, so that we can assess how communities form as we become more, or less, relaxed about the distance required to regard treatments as close. In our code, our default is to explore the 20%, 40%, 80% and 80% quantiles, though any quantiles can be chosen. With three measures of distance, and four thresholds, our default results in examining 12 community structures. We have found this to be an adequate, but not overwhelming, number of possibilities to explore. Our code takes each quantile in turn and graphically displays the resulting network, with the optimal community structure, for all three types of distances simultaneously. Examples are shown for our real example below. In this way we can quickly and easily assess which treatments are estimated to have similar efficacy (using our first measure of distance), which parts of the network are better identified (using our second measure of distance) and for which pairs treatments the statistical significance of their difference is weakest and strongest (using our third measure of distance).

Full, detailed code for undertaking the visualisation is provided in the Additional file 1. However, the method can be summarised as a three-step process:

- Step 1: Use the estimates derived from any meta-analysis estimation method to create a distance matrix.
- Step 2: Using some threshold, normalise the distance matrix to create an adjacency matrix.
- Step 3: Utilise community detection methods to uncover the optimal group structure and display this structure on a network diagram.

### The second visualisation method: Taking uncertainty into account

When using community detection algorithms, vertices are either connected or they are not. In most network analysis applications this makes sense. However there is statistical uncertainty in the estimated treatment effects that are used to calculate the first of our distance measures and this is not taken into account when using our first visualisation method. In other words, we do not really know if treatments are close enough to be considered connected when using our first measure, because we only have estimates of their true values.

In fact, there is also statistical uncertainty in the standard errors used to calculate our second and third distance measures, but it is common-practice in meta-analysis to take all variance components as known when performing the pooling [16*,*^17^*]. This is a reasonable approximation in large samples, where normal approximations with ‘known’ variance are often used for the estimated treatment effects. Hence refraining from taking into account the uncertainty in the standard errors is of much less concern. Furthermore the meaning of a p-value is ‘the probability that the chosen test statistic would have been at least as large as its observed value if every model assumption were correct, including the test hypothesis’ [*20]. Although assumptions are needed to calculate this probability, if the standard errors are treated as fixed then there is no uncertainty in this calculation. Ignoring the statistical uncertainty in the fitted model is not a serious source of concern when using our second and third measures of distance, but it is for the first.

In order to take into account this uncertainty when using our first distance measure, a parametric bootstrapping procedure was adopted. Here we simulate many realisations from the multivariate distribution $N\left (\hat {\boldsymbol {\delta }}, \hat {\boldsymbol {V}}\right)$, where the necessary quantities for our artificial example are given in Eq. (1). Realisations such as these were also used by White et al. [21] for performing approximate classical ranking (see their ‘Ranking in the consistency model’ section). Then for each realisation from $N\left (\hat {\boldsymbol {\delta }}, \hat {\boldsymbol {V}}\right)$ we use the same procedure as described above for our first measure of distance (including the use of a particular threshold) but we instead use the random realisation as the estimated treatment effects. This results in a different community structure for each simulated realisation. We calculate the proportion of simulated realisations that result in each treatment pair being placed in the same community, and display these proportions using a heat map. We emphasise that these proportions do not have a probabilistic interpretation such as estimating the probability that each treatment belongs to the same community. For this a Bayesian approach, where the likelihood used in the analysis is the probability distribution of community structures, would be required. We leave this possibility as an avenue for further work. Heat plots have been suggested previously to show the ranking of treatments [22] but the plots suggested here are conceptually different because are used to identify communities of treatments with similar or different estimated efficacies.

### Weighted and unweighted approaches

For both methods, there are two approaches based on whether the user wishes to use weighted or unweighted adjacency matrices – that is, whether the remaining connections should be given identical weights or not. In the unweighted case, relative effect estimates, standard errors and *p* values beyond a given threshold are deemed unconnected in the adjacency matrices – that is, given a value of zero. Otherwise, they are deemed connected – that is, given a value of one.

We also include the option of using weighted adjacency matrices. In the initial adjacency matrices, all vertices are either connected (indicated by a one) or not connected (indicated by a zero). However, more generally, connected vertices can be indicated in adjacency matrices using any positive value, where larger entries indicate stronger connections. A simple way to produce weighted adjacency matrices from distance matrices is by taking the reciprocal of all off-diagonal entries of a distance matrix **D** that are less than or equal to the threshold and taking all other entries to be zero. However this choice or taking the reciprocal is somewhat arbitrary; the reciprocal function serves to give greater weights to treatments that are closer together (and do not exceed the threshold) but any other decreasing function that transforms positive values in this way could also be used for this purpose.

In summary, in the weighted case, existing connections between treatments are given a weight, where a greater weight indicates a stronger connection. In the case of estimates and standard errors, the reciprocal is taken. In the case of *p* value, the reciprocal of the complement is taken.

## Results

### Example: Osteoarthritis of the knee

The fitted model that we will use to illustrate our visualisation methods is from an example involving a collection of studies comparing treatments for osteoarthritis of the knee [23*]. The data comprises 87 studies comparing a total of 22 treatments. The measurement used to compare the treatments is the standardised mean difference of pain at trial end. The results were obtained using a random-effects model that allows for random effects in both the between-study heterogeneity and the inconsistency [*24], and the dataset has been analysed previously by Jackson *et al* [15,16]. Specifically, we will use the inconsistency model fitted using the method of moments from Jackson *et al* [16] (see their Table 3) to illustrate our methods. The primary inferences from this fitted model is shown in Table 1, where the upper triangle shows the relative treatment effects (negative estimates indicate treatment benefit) and the lower triangle shows the corresponding standard errors. The absolute values of the upper triangle are the distances contained in the matrix ***D***_1_ and the values in the lower triangle are the distances contained in ***D***_2_. Table 1 nicely highlights the difficulty in visualising fitted models for network meta-analysis when many treatments are present; Figs. 1 and 2 assist with this but our methods are intended to help us better understand the implications of fitted models such as this one.

**Table 1:** Relative treatment effects (upper triangle) and standard errors (lower triangle) for osteoarthitis of the knee dataset

	A	B	C	D	E	F	G	H	I	J	K	L	M	N	O	P	Q	R	S	T	U	V
A	–	0.04	0.60	-0.78	-0.46	-0.15	-0.59	-0.37	-0.03	-0.01	-0.28	-0.35	-0.25	-0.25	-1.11	-0.25	-0.29	0.46	-0.70	0.01	-0.78	-0.63
B	0.20	–	0.56	-0.82	-0.50	-0.19	-0.63	-0.41	-0.07	-0.05	-0.32	-0.39	-0.30	-0.29	-1.15	-0.29	-0.33	0.41	-0.75	-0.03	-0.82	-0.67
C	0.32	0.32	–	-1.37	-1.06	-0.75	-1.19	-0.97	-0.63	-0.61	-0.88	-0.95	-0.85	-0.85	-1.71	-0.85	-0.89	-0.14	-1.30	-0.59	-1.38	-1.23
D	0.16	0.24	0.35	–	0.32	0.63	0.18	0.40	0.75	0.77	0.49	0.43	0.52	0.52	-0.34	0.53	0.49	1.23	0.07	0.78	-0.01	0.15
E	0.25	0.19	0.37	0.28	–	0.31	-0.14	0.09	0.43	0.45	0.17	0.11	0.20	0.20	-0.65	0.21	0.17	0.91	-0.25	0.47	-0.32	-0.17
F	0.46	0.50	0.56	0.49	0.53	–	-0.44	-0.22	0.12	0.14	-0.13	-0.20	-0.11	-0.10	-0.96	-0.10	-0.14	0.60	-0.56	0.16	-0.63	-0.48
G	0.22	0.30	0.38	0.27	0.33	0.51	–	0.22	0.57	0.59	0.31	0.25	0.34	0.34	-0.52	0.35	0.31	1.05	-0.11	0.60	-0.19	-0.03
H	0.11	0.20	0.31	0.19	0.26	0.47	0.24	–	0.34	0.36	0.09	0.02	0.12	0.12	-0.74	0.13	0.08	0.83	-0.33	0.38	-0.41	-0.26
I	0.30	0.28	0.40	0.33	0.33	0.55	0.37	0.30	–	0.02	-0.26	-0.32	-0.23	-0.22	-1.08	-0.22	-0.26	0.48	-0.68	0.04	-0.75	-0.60
J	0.35	0.29	0.43	0.37	0.34	0.58	0.41	0.35	0.40	–	-0.28	-0.34	-0.25	-0.24	-1.10	-0.24	-0.28	0.46	-0.70	0.02	-0.77	-0.62
K	0.29	0.35	0.43	0.33	0.38	0.54	0.36	0.31	0.42	0.45	–	-0.06	0.03	0.03	-0.83	0.04	-0.00	0.74	-0.42	0.29	-0.50	-0.34
L	0.26	0.33	0.41	0.30	0.36	0.53	0.31	0.28	0.40	0.44	0.39	–	0.09	0.09	-0.76	0.10	0.06	0.80	-0.36	0.35	-0.43	-0.28
M	0.23	0.29	0.39	0.18	0.33	0.52	0.32	0.25	0.37	0.41	0.37	0.35	–	0.00	-0.86	0.01	-0.03	0.71	-0.45	0.26	-0.53	-0.37
N	0.37	0.35	0.42	0.38	0.39	0.59	0.43	0.37	0.40	0.45	0.47	0.45	0.42	–	-0.86	0.01	-0.04	0.71	-0.45	0.26	-0.53	-0.37
O	0.48	0.45	0.50	0.50	0.48	0.67	0.53	0.48	0.52	0.53	0.56	0.54	0.53	0.55	–	0.86	0.82	1.57	0.41	1.12	0.33	0.48
P	0.36	0.30	0.44	0.38	0.35	0.59	0.42	0.36	0.41	0.42	0.46	0.44	0.42	0.46	0.54	–	-0.04	0.70	-0.46	0.25	-0.53	-0.38
Q	0.30	0.32	0.42	0.34	0.36	0.55	0.37	0.31	0.41	0.43	0.42	0.40	0.38	0.45	0.55	0.44	–	0.74	-0.42	0.30	-0.49	-0.34
R	0.55	0.58	0.63	0.57	0.60	0.72	0.59	0.56	0.63	0.65	0.62	0.60	0.59	0.66	0.73	0.66	0.62	–	-1.16	-0.45	-1.24	-1.08
S	0.32	0.29	0.42	0.35	0.34	0.56	0.39	0.32	0.39	0.41	0.43	0.41	0.39	0.44	0.53	0.42	0.42	0.64	–	0.71	-0.08	0.08
T	0.32	0.24	0.40	0.34	0.31	0.56	0.38	0.31	0.37	0.38	0.43	0.41	0.38	0.42	0.51	0.39	0.41	0.63	0.38	–	-0.79	-0.63
U	0.57	0.53	0.62	0.58	0.56	0.73	0.61	0.57	0.60	0.60	0.64	0.62	0.61	0.63	0.70	0.61	0.62	0.79	0.60	0.58	–	0.15
V	0.22	0.19	0.34	0.25	0.26	0.51	0.31	0.22	0.30	0.34	0.36	0.34	0.30	0.36	0.48	0.36	0.35	0.59	0.33	0.31	0.56	–

For relative treatment effects, a positive value indicates effect of row treatment is superior to column treatment. A: standard care; B: placebo; C: no medication; D: acupuncture; Ebalneotherapy; F: braces; G: aerobic exercise; H: muscle exercise; I: heat treatment; J: insoles; K: tai chi; L: weight loss; M: sham acupuncture; N: ice/cooling; O: interferential; P: laser; Q: manual; R: neuromuscular electrical stimulation (NMES); S: pulsed electrical stimulation (PES); T: PEMF; U: static magnets; V: transcutaneous electrical nerve stimulation (TENS)

**Fig. 5:**
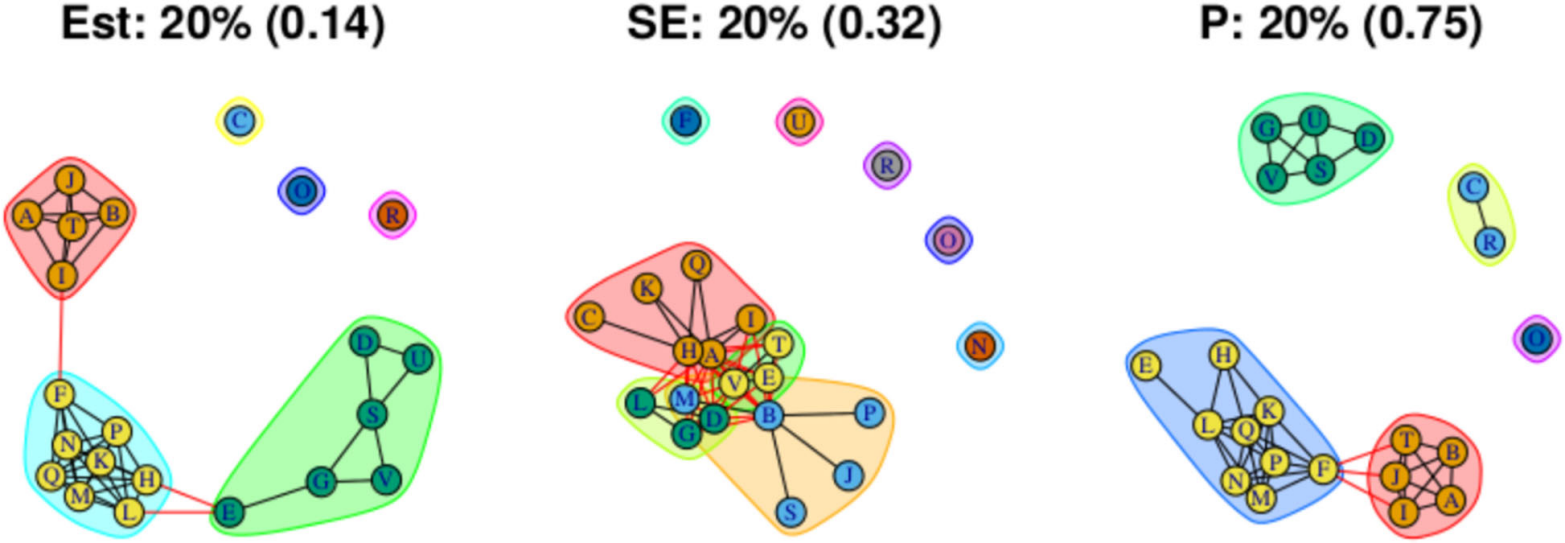
Groupings for example dataset (unweighted), using first method with threshold at 20%

**Fig. 6:**
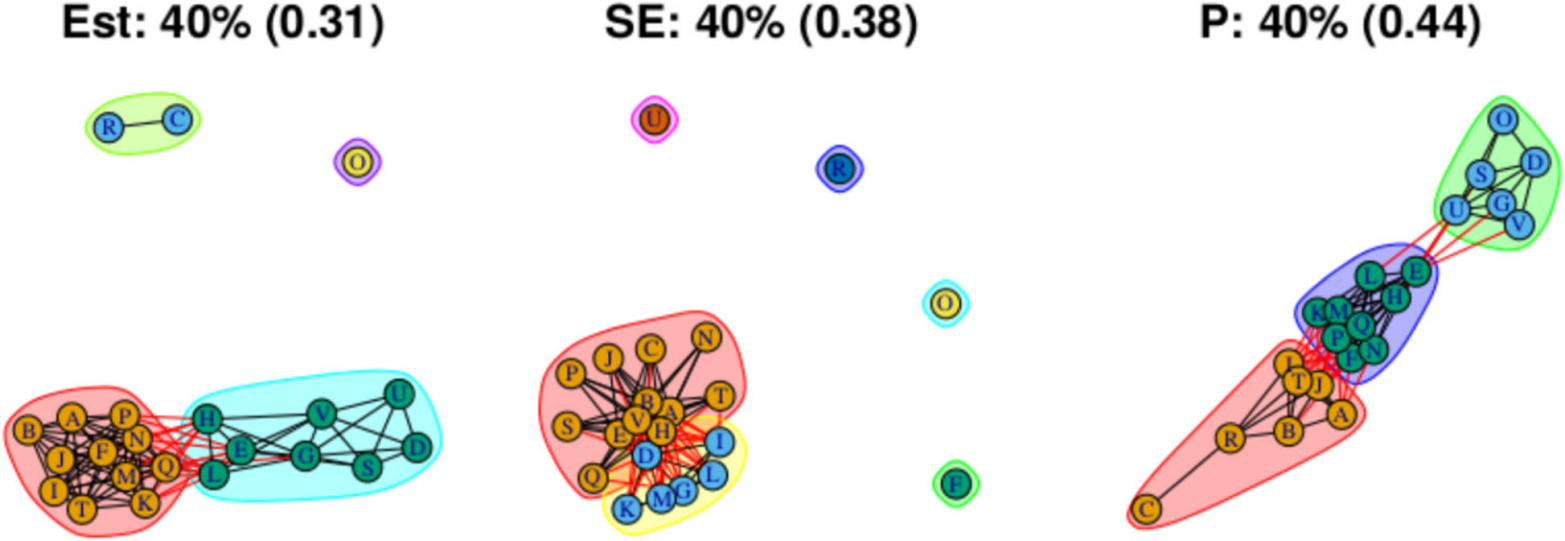
Groupings for example dataset (unweighted), using first method with threshold at 40%

**Fig. 7:**
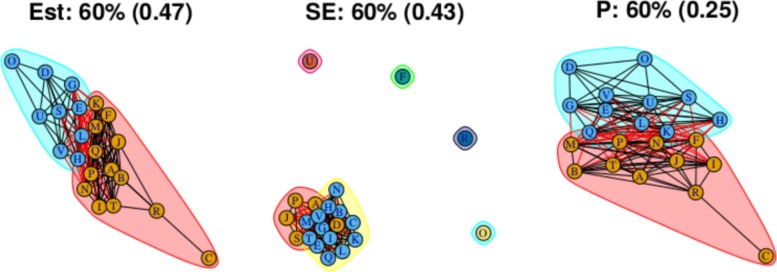
Groupings for example dataset (unweighted), using first method with threshold at 60%

**Fig. 8:**
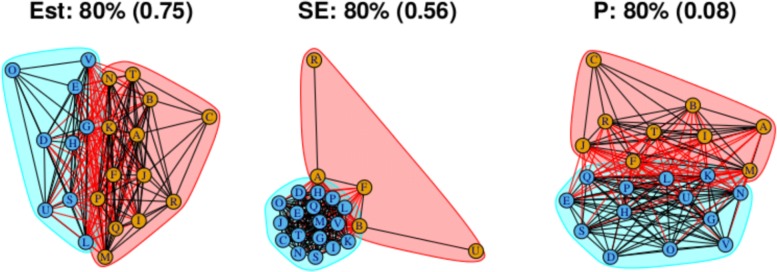
Groupings for example dataset (unweighted), using first method with threshold at 80%

**Fig. 9:**
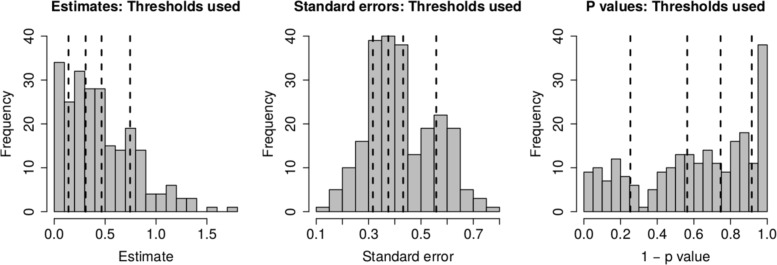
Historgrams showing thresholds for 20%, 40%, 60% and 80% quantiles for each distance measure

**Fig. 10:**
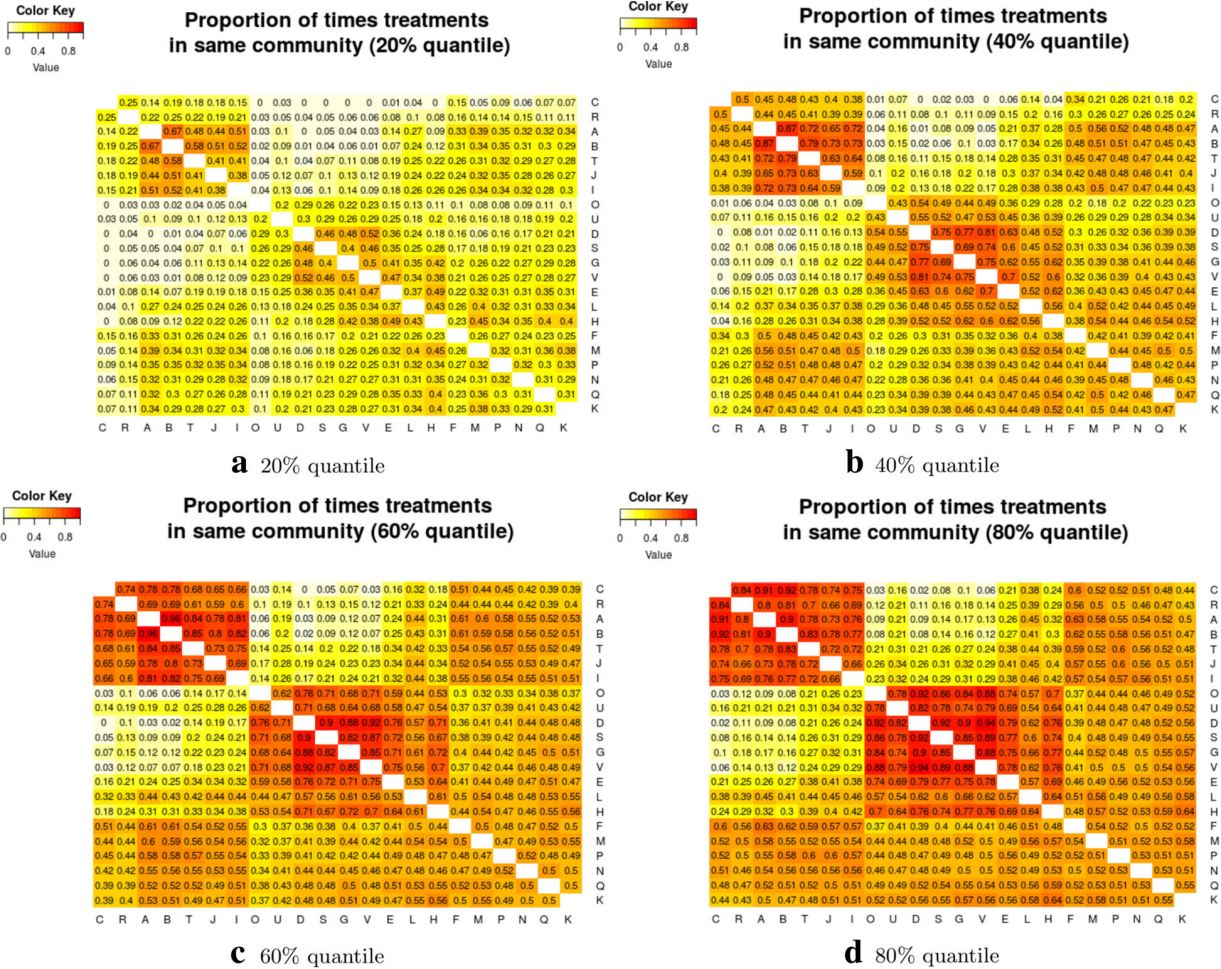
Relative treatment effect estimates, bootstrap method: Heatmaps showing the proportion of times each pair of treatments was in the same community, with regards to treatment effect. **a** 20% quantile. **b** 40% quantile. **c** 60% quantile. **d** 80% quantile

As stated above, the output for the first method comprises three plots for every threshold used – one each for the (absolute) treatment effect estimates, the standard errors and the (the complement of) the *p* values – and so employing four thresholds (20%, 40%, 60%, 80% quantiles) results in 12 plots. These plots are shown in Figs. 5, 6, 7 and 8. In all figures, (absolute) relative effect estimates, standard errors and (the complement of) the *p* values are displayed in the left, middle and right plots respectively. The values of the quantiles for each characteristic – used for the first visualisation method – are shown in Table 2, and in the context of the observed distributions of the distance measures in Fig. 9. These are the 20%, 40%, 60% and 80% quantiles of the three distance measures. For example, the 40% threshold of the (absolute) estimated relative effects estimates in Table 1 is 0.31 (Table 2). Having determined suitable thresholds, adjacency matrices can be calculated and community detection algorithms applied in the way described above. The output of the second method comprises one heat map per threshold used, resulting in four heat maps. These four plots are shown in Fig. 10.

**Table 2:** Thresholds used in the visualisation of the model fitted to the osteoarthitis of the knee dataset (first method)

Quantile	Characteristic		
	(Absolute) Effect estimate	Standard error	(Complement of) *P* value
20%	0.14	0.32	0.25
40%	0.31	0.38	0.56
60%	0.47	0.43	0.75
80%	0.75	0.56	0.92

We will examine each of the three distance measures in turn, including a comparison of the output of the first and second methods for the (absolute) relative effect estimates. We provide output for both the weighted and unweighted approaches, but focus on the unweighted approach, briefly describing the results of the weighted approach in terms of its similarity to the unweighted approach. For the heat map used in the second method, the accompanying code can reorder the treatments using *R*’s default hierarchical clustering method. This reordering takes place for the first threshold and resulting plot then fixed for subsequent plots, allowing easier comparison of heat maps across thresholds. We obtain results using both visualisation methods.

### Treatment effect estimates

Figure 5 (left) shows the community structure for treatment effect estimates obtained using a threshold placed at the 20% quantile. There are three distinct, large communities, with three more single treatment communities. Edges that exist across, rather than within, communities are shown in red. The treatments A, B, I, J and T are contained in one community, with a single edge to a second community, comprising the treatments F, H, K, L, M, N, P and Q. This second community is linked by two edges to a third community comprised of treatments D, E, G, S, V and U. This community structure is reflected in the corresponding output from the second method, shown in Fig. 10a, where, the “ABIJT” community can be seen, as can the “DEGSVU”. Many of the weakest connections, those with the smallest proportions of times in the same community, come from pairing one treatment each from these communities. The single-treatment communities are treatments C, O and R. The poorest performing treatment is C, followed by R (Table 1). The community containing placebo and standard care, the ABIJT community, contains other poorly performing treatments. The treatment with the greatest efficacy is O. The DEGSVU community contains the next-best performing treatments and has no direct links to any treatments from the community containing the poorest treatments. The centre “FHKLMNPQ” community contains treatments with efficacy between these two communities.

The community structures obtained from a threshold using the 40% quantile are shown in Fig. 6. For the relative treatment effect estimates (left figure), there are now two large and two small communities: Treatments from the middle community in the previous figure have been absorbed into the two more extreme communities on either side, with the less effective community (ABIJT) now containing treatments F, K, M, N, P and Q, and the better performing community (DEGSVU) now containing treatments E, H and L. The treatments with the poorest efficacy, C and R, have been combined into a single community. Figure 10b, the corresponding output from the second method, also shows these changes in the community structure, with two large communities beginning to appear.

Using the 60% quantile (Fig. 7), all treatments have now been absorbed into one of two large communities, with the treatments with the greatest efficacy – D, O, U, and so on at one end of the network in one community, and those with the lowest efficacy – C and R – at the opposite end of the network in another community. However, there are also many connections across the two communities, suggesting that there is similarity in relative treatment effect estimate among certain treatments across the communities. The output from the second method, shown in, Fig. 10c, is somewhat in agreement, with some very high proportions of connections between treatment pairs in the extremities of the communities, very low proportions of connections regarding treatment pairs at opposite ends of the community structure, and similar proportions of connections across most other treatment pairs. This suggests that there may be high similarity of effect estimates within a subset of each community, low similarity across those two subsets, and moderate similarity between treatments in the centre of the community structure when compared with any other treatment.

The output from the final threshold used, the 80% quantile, is shown in Fig. 8. There remain two communities with an increased number of connections across them. From the second method with the threshold set at the 80% quantile, Fig. 10d, we can also identify two main communities. However, the output does not suggest that the two communities necessarily encompass all treatments. Like the heat map in Fig. 10c, there are high proportions of connections between two smaller communities: One containing treatments A, B, C, I, J, R and T, and the other containing treatments D, E, G, H, L, S, U, and V.

To summarise, the results using the 60% and 80% thresholds suggest that, if we are to require larger estimated effects to indicate worthwhile treatment benefit, then there is some evidence of the presence of two communities of treatments. The treatments in the first of these communities possess similar effectiveness to standard care but those in the second community (DEGHLOSUV) appear to be more effective. However the other quantiles indicate that the situation is more complicated, in particular if we consider smaller estimated effects to be worthwhile then multiple communities of treatments appear. The heat plots further suggest that the discrete nature of any community structure is likely to be considered overly simplistic. Despite the difficulties presented by this challenging example, our new results add considerable insight, for example they suggest that patients who are in considerable pain and require the greatest potential pain relief should perhaps consider one of the treatments in the second community that our methods have identified.

### Standard errors

Standard errors and *p* values are examined using the first method only. The community structure obtained using the 20% threshold is shown in Fig. 5 (centre) and is comprised of four small communities that are closely connected and five unconnected communities each containing one treatment. Those treatments are F, N, O, R and U. Connections between treatments indicate low standard error estimates and thus well-identified relative effect estimates. The single-treatment communities indicate a lack of information regarding these effect estimates compared to others. Output from thresholds at the 40% and 60% quantiles (Figs. 6 and 7, centre) are similar, containing four single-treatment communities and two very closely connected communities. The output obtained from using a threshold at the 80% quantile (Fig. 8, centre) shows one large community containing most of the treatments, with a small second community containing three of the treatments that were previously in communities on their own. Note that the previously “individual” communities become connected to the rest of the treatments at different points of the network, including U to B and R to A; treatments U and R each only appear in one study in the network, being compared to B and A respectively in those studies, thus the connections make intuitive sense.

The overall conclusion is that, while the relative effects of most treatment pairs have relatively similar levels of identifiability, there are some treatments – F, O, R, and U (and to some extent, N) – that are less well identified. For these particular treatments the precision of any treatment comparison is low when compared to the rest of the network. Figure 1 indicates that this might be anticipated, because there are very few direct connections involving these treatments, but there are other treatments for which this is also the case whose treatment effects are better identified. Our methods have therefore successfully highlighted the parts of the network that are less well identified from the fitted model. Our finding that, relative to the rest of the network, four or five of the treatments are so poorly identified is at best much less obvious unless we use our new visualisation methods.

### *P* values

For this distance measure, a connection between a pair of treatments indicates that the *p* value is large, and so there is little evidence of a difference between the treatments when both effect size and standard error are taken into account. The community structure obtained using the 20% threshold is shown in Fig. 5 (right), and shows five communities of differing sizes, with few connections across them. This set of structures resembles that of the treatment effect estimates (Fig. 5, left), which is to be expected. Note that the value of the threshold is 0.25, meaning that connections exist between treatment pairs for which the relative effect estimate has a *p* value of greater than 1−0.25=0.75. Using the 40% threshold results in three communities, with two communities connected only to a central community and not to one another. The membership of these communities resembles the community structure for the effect estimates using the 20% quantile (in Fig. 5 (left)), where the treatment pairs with the greatest differences in estimates are far apart in terms of the network structure. However, here, the distance is based on *p* value rather than solely the estimates. The output from using the 60% and 80% thresholds are similar to one another, showing community structures with two large communities that also show the treatment comparisons with the smallest *p* values as being far apart.

The overall conclusions when examining the *p* values is similar to when examining the treatment effects above; the presence of two communities is apparent but again our visualisation devices indicate that the situation is somewhat more complicated than this.

### Weighted results

**Fig. 11:**
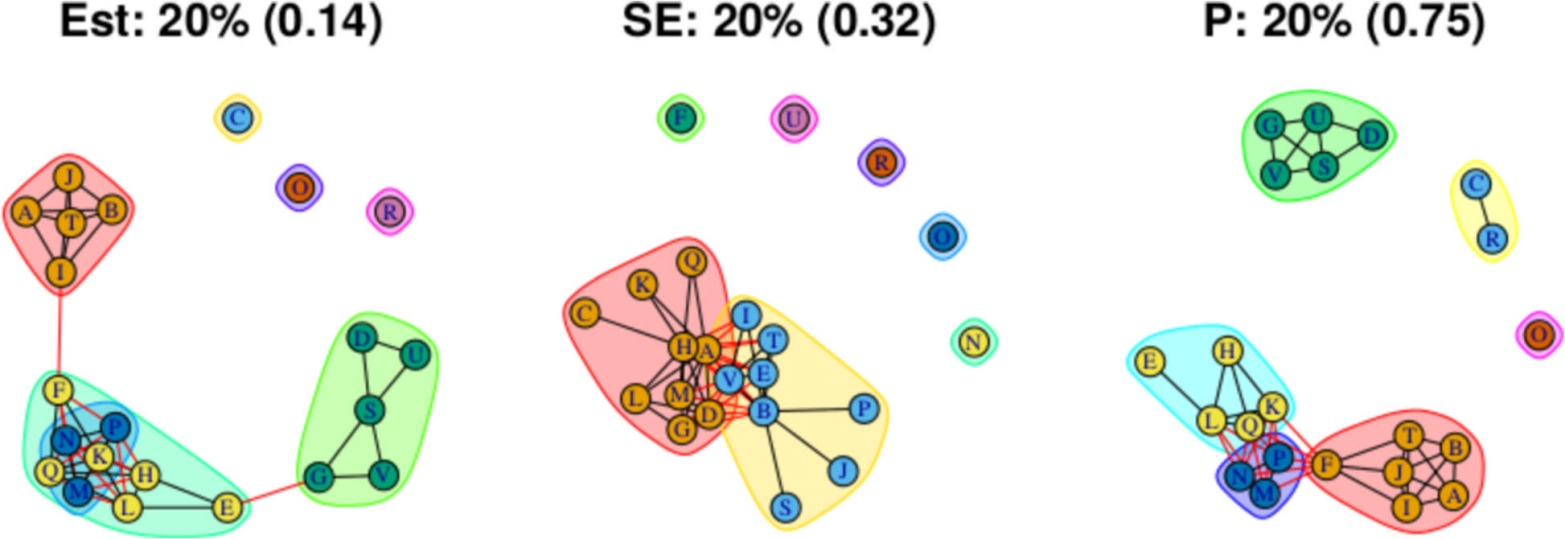
Groupings for example dataset (weighted), using first method with threshold at 20%

**Fig. 12:**
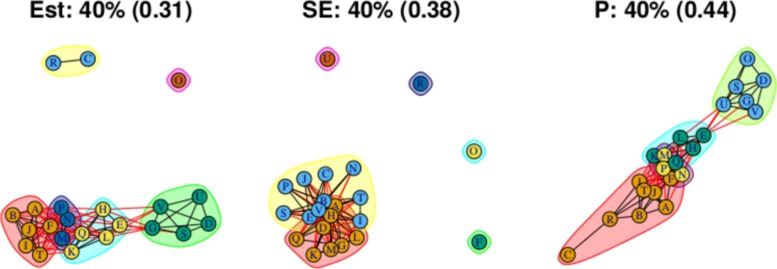
Groupings for example dataset (weighted), using first method with threshold at 40%.

**Fig. 13:**
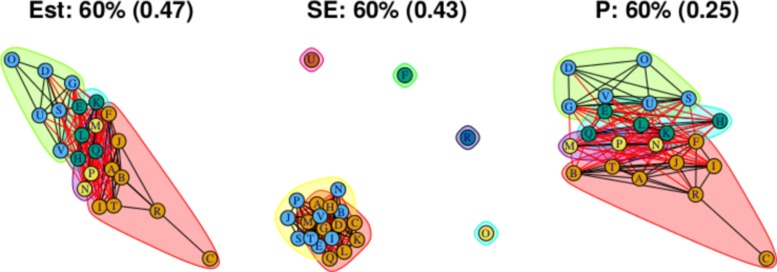
Groupings for example dataset (weighted), using first method with threshold at 60%

**Fig. 14:**
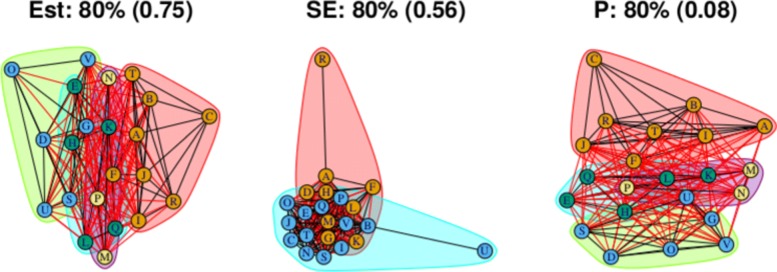
Groupings for example dataset (weighted), using first method with threshold at 80%

The community structures created for all three distance measures and and four specified quantiles using the weighted approach are shown in Figs. 11, 12, 13 and 14. These are the weighted equivalent to the community structures in Figs. 5, 6, 7 and 8. The communities found using the weighted approach are broadly similar to those found using the unweighted approach. The main difference in this example is that the weighted approach tends to create one or two more communities when using the distance measures of treatment effect estimate and *p* value. In general, we find that the weighted approach makes the boundaries between communities identified by the unweighted approach less clear.

## Discussion

We have developed two new, and closely related, methods for visualising the implications of fitted models for network meta-analysis. Our methods use algorithms for community detection, a concept originating in network analysis, to group treatments using insightful criteria.

We have explained how weighted and unweighted adjacency matrices may be used in conjunction with both our methods. In the weighted case, we take the reciprocal of the distance measure. However, the reciprocal gives very considerable, and often excessive, weight to treatments that appear (perhaps by chance) to be very close together. We have not therefore found the use of weighted adjacency matrices very helpful when visualising models for network meta-analysis. Other approaches for calculating weighted adjacency matrices may prove more satisfactory than the approach proposed here, and we leave this as a potential avenue for further work.

Our distance measure based on standard errors highlights which parts of the network are least well identified. This is not necessarily obvious from standard statistical output and so we regard this as an important contribution of our work. However, our methods do not explain why some parts of the network may be less well identified. This is an important subsequent question to address. For example, a particular set of comparisons may be poorly identified because there is little or no direct evidence for them, for example because these combinations of treatments could be less suitable for the same types of patient. Alternatively, this could be because these treatments are a combination of older and newer treatments, and so have not been directly compared for historic reasons. Having used our methods to determine which parts of the network are less well identified, additional considerations will be required to determine why this is the case. Another issue is that our methods based on distances defined by estimated effect sizes may be affected by publication biases. However our methods could be used in conjunction with methods and models that adjust for publication bias [25] and we leave methods that form communities whilst adjusting for publication biases as an avenue for future work.

In this paper we implement the three distance measures described above. However alternative measures of distances between treatments may also be useful and we strongly encourage the consideration of other possibilities. In particular, incorporating distance measures that measure inconsistency [26*] within the network is one exciting possibility. However inconsistency is usually conceptualised as differences between the results from studies that include different combinations of treatments, rather than differences between the treatments themselves [*21]. Hence developing distance measures that measure inconsistency in the network is not straightforward, but one possibility is to define a distance that is based on the differences between estimated treatment effects under models that assume consistency and allow this assumption to be relaxed, and thus measuring the impact of inconsistency in the network. Other possibilities include measuring statistical significance in other ways, for example basing this measure on test statistics directly rather than *p*-values; although we use empirical quantiles of distances to determine community structures, the use of test statistics rather than p-values will make a difference if the weighted version of our methodology is used. Furthermore distance measures based on p-values and/or test statistics for alternative hypothesis tests, for example that test for clinical rather than statistical significance, may also be of interest.

In many respects the the authors prefer the second method because it explicitly allows for the uncertainty in the fitted model in a statistically principled way. However the first method allows us to simultaneously visualise multiple characteristics of the fitted model. This allows the user to easily understand not only the relative magnitudes of the effects, but also their degree of precision and statistical significance. Thus, both methods are valuable tools to understand the results of a network meta-analysis. When fitting a Bayesian model for network meta-analysis we could use draws from the posterior distribution when using our second method, instead of the parametric bootstrapping procedure that we have proposed here, in order to avoid using normal approximations for the posterior distribution of the estimated treatment effects. When fitting a Bayesian model for network meta-analysis we could use draws from the posterior distribution when using our second method, instead of the parametric bootstrapping procedure that we have proposed here, in order to avoid using normal approximations for the posterior distribution of the estimated treatment effects. The normal approximations required by the bootstrap method are not always very accurate, especially in situations where the outcome data are binary the event is rare, or if there are just a few small studies. See Jackson and White [27] for a full discussion of this and related issues.

A limitation of the current work is that our methods for community detection use only the concept of modularity maximisation. There are approaches to community detection other than modularity maximisation, and the use of these other approaches could be examined. An issue is that there may exist disparate sets of community groupings, which result in qualitatively different conclusions, each with modularity close to the maximum. This possibility is not revealed by our methods and strategies to assess this possibility would embellish our ideas. However, this issue is partly ameliorated by the use of a range of thresholds, which in any case provide the analyst with a range of community structures to consider. It may be that our methods are best used informally, in order to help analysts explore the implications of their fitted model, but we would also encourage analysts to consider using them more formally by providing plots using our methods in published reports and papers.

Regarding computation time, the results were obtained using a computer containing an i7-4790 processor and 16 gigabytes of RAM. Computation time for the example dataset (22 treatments, four quantiles) using the first method was just a few seconds. For the second method, the computation time was around three minutes. As stated above, the time taken to calculate the modularity for every possible grouping increases exponentially with the number of nodes, and so computation time will be less for most network meta-analysis datasets.

## Conclusions

In summary, we have presented two new methods for visualising fitted models for network meta-analysis, so that their implications may be better understood. We have demonstrated that our methods add considerable insight when applied to model that was previously fitted to a challenging real network meta-analysis dataset. Our methods were developed using the software *R*. Full computing code is provided in the Additional file 1, where the code is explained in detail. The example dataset is also provided in these Additional file 1, along with all output.

## Additional file

Additional file 1 Supplementary material: Grouping treatments into communities. This file contains: An in-depth explanantion regarding how the treatments are grouped into communities; Full R code and an explanation of how it may be used in practice; The example data used, and R code that reproduces the figures in the main manuscript and this document, and figures showing the output of both methods, using both unweighted and weighted approaches. (DOCX 1896 kb)

## Abbreviations

SUCRA: Surface under cumulative ranking curve
