# Supplementary material for: Two new approaches for the visualisation of models for network meta-analysis
Source: BMC Med Res Methodol. 2019 Mar 18;19:61. doi: 10.1186/s12874-019-0689-9 (PMC6423884; doi:10.1186/s12874-019-0689-9)
Supplement: Supplementary file 1 — Supplementary material: Grouping treatments into communities. This file contains: An in-depth explanantion regarding how the treatments are grouped into communities; Full R code and an explanation of how it may be used in practice; The example data used, and R code that reproduces the figures in the main manuscript and this document, and figures showing the output of both methods, using both unweighted and weighted approaches. (DOCX 1896 kb) [file 12874_2019_689_MOESM1_ESM.docx]

**Supplementary material: Grouping treatments into communities**

**Modularity calculation**

As an example of modularity calculation, we calculate the modularity for both community structures in Figure 4. We require the degree of each vertex *k_i_, i*=1, 2, 3, 4, and the number of edges, *m*. As stated in the main paper, these values are k_1_=1, k_2_=3, k_3_=2, k_4_=2, m=4. Note that these values are characteristics of the network, and are independent of community structure. For the community structure in Figure 4a, the observed number of edges lying within communities is O_1_=3. To calculate the expected number of edges lying within communities, we take the 1/2*m* term outside the summation in Equation (7) of the main paper so that

$$\begin{matrix} E_{1}=\frac{1}{2}\frac{1}{2m}\left[k_{1}k_{1}+k_{2}k_{2}+k_{2}k_{3}+k_{2}k_{4}+k_{3}k_{2}+k_{3}k_{3}+k_{3}k_{4}+k_{4}k_{2}+k_{4}k_{3}+k_{4}k_{4}\right] \\ =\frac{1}{2}\frac{1}{2\cdot4}\left[1\cdot13\cdot3+3\cdot2+3\cdot2+2\cdot3+2\cdot2+2\cdot2+2\cdot3+2\cdot2+2\cdot2\right] \\ =\frac{50}{16}=3.125. \end{matrix}$$

For clarity, terms that do not contribute to the calculation have been omitted, that is, all *k_i_ k_j_* terms where *C_i_ C_j_*. Subtracting *E_1_* from *O_1_* and dividing by *m* gives the modularity of the network and community structure: *Q_1_* = *(O_1_–E_1_)/m* = (3-3.125)/4 = -0.03125.

Similarly, for the community structure in Figure 4b, the number of edges inside communities is *O_2_*=2, and the number of expected edges inside communities is

$$\begin{matrix} E_{2}=\frac{1}{2}\frac{1}{2m}\left[k_{1}k_{1}+k_{1}k_{2}+k_{2}k_{1}+k_{2}k_{2}+k_{3}k_{3}+k_{3}k_{4}+k_{4}k_{3}+k_{4}k_{4}\right] \\ =\frac{1}{2}\frac{1}{2\cdot4}\left[1\cdot1+1\cdot3+3\cdot1+3\cdot3+2\cdot2+2\cdot2+2\cdot2+2\cdot2\right] \\ =\frac{32}{16}=2 \end{matrix}$$

and so the modularity is *Q_2_=(O_2_–E_2_)*/4 = 0. Thus the second community structure, shown in Figure 4b, has a slightly greater modularity than the first (0 vs. -0.03125), and as stated in the main paper, we would take this grouping to be the better representation of the network. The modularity for both community structures are similar, as the networks are simple and there is only one difference between them, with just one treatment, B, transferring from one community (containing C and D) to another (containing A). With larger, less trivial networks, potential differences in modularity are greater.

**R code**

The R function cd (“community detection”) below allows analysts to visualise the similarity of treatments in a network, using one of two related methods. As described in the main body of the paper, the first method involves creating one or more sets of three adjacency matrices (based on estimated effects, standard errors and p values) from the data directly, then grouping the treatments into communities based on those matrices and examining those communities using plots.

The second method involves creating a series of adjacency matrices (based on estimated effects only) from bootstrap replications, and again grouping the treatments, resulting in a series of groupings, one for each replication. This series of groupings is then aggregated by way of a heat map. Both methods involve examining plots corresponding to groupings made with respect to user-specified thresholds.

Regardless of the method chosen, adjacency matrices are used. The adjacency matrices describe the connections between the treatments. In the sense in which we are comparing the treatments (whether using only estimated relative effects, or additionally their standard errors and p values), every treatment is connected to every other treatment: Each pair of treatments has an associated estimated relative effect, along with an associated standard error and p value. This makes community detection more difficult, therefore we remove the weakest connections before undertaking community detection. This is done by choosing a threshold (or series of thresholds) beyond which treatments are deemed to be unconnected – for example, a large relative effect estimate, standard error or small p value. At this stage, we use the compliment of the p value (that is, 1-p) to be able to make the overarching statement that larger values imply more dissimilar treatments. The threshold is a quantile of (say) the relative effect estimates. We encourage the use of a range of quantiles – for example, quartiles or tertiles – for a fuller understanding of how the treatments relate to one another.

For each method, there are two approaches, based on whether the user wishes to use weighted or unweighted adjacency matrices – that is, whether the remaining connections should be given identical weights or not. In the unweighted case, relative effect estimates, standard errors and p values beyond a given threshold are deemed unconnected in the adjacency matrices – that is, given a value of zero. Otherwise, they are deemed connected – that is, given a value of one. This is the approach presented in the manuscript, and is preferred by the authors.

We also include the option of using weighted adjacency matrices. In the initial adjacency matrices, all vertices are either connected (indicated by a 1) or not connected (indicated by a 0). However, more generally, connected vertices can be indicated in adjacency matrices using any positive value, where larger entries indicate stronger connections. A simple way to produce weighted adjacency matrices from distance matrices is by taking the reciprocal of all off-diagonal entries of a distance matrix D that are less than or equal to the threshold and taking all other entries to be zero. However this choice or taking the reciprocal is somewhat arbitrary; the reciprocal function serves to give greater weights to treatments that are closer together (and do not exceed the threshold) but any other decreasing function that transforms positive values in this way could also be used for this purpose. Furthermore the reciprocal gives very considerable, and often excessive, weight to treatments that appear (perhaps by chance) to be very close together. We have not therefore found the use of weighted adjacency matrices very helpful when visualising models for network meta-analysis. In summary, in the weighted case, a threshold is again used, and again unconnected treatment pairs are given a value of zero in the adjacency matrices. However, connections between treatments are now given a weight, where a greater weight indicates a stronger connection. In the case of estimates and standard errors, the reciprocal is taken. In the case of p value, the reciprocal of the compliment is taken.

To create these adjacency matrices, the data required are a vector of treatment effect estimates relative to a single reference treatment, which has length t-1 where t is the number of treatments (argument est below), and the (square) covariance matrix of dimension t-1 (argument cov below). A small initial function, fns, within the cd function uses these data to form matrices of (absolute) effect size estimates, their standard errors and associated (compliment of the) p values. These matrices are the basis of the adjacency matrices that are then used to group the treatments.

The creation of the matrix of relative treatment effect estimates is not obvious. Define the true relative effect of some treatment B against a reference treatment A, i.e. A –B, as $\delta^{AB}$. Now define the vector of estimated treatment effects (relative to reference treatment A) as $\hat{\boldsymbol{\delta}}=\left[ \hat{\delta}^{AB} \hat{\delta}^{AC} \hat{\delta}^{AD}\cdots\hat{\delta}^{At-1} \right]$, where *t* equals the total number of treatments. The entries of this vector are known as the *basic parameters*, and linear combinations of these basic parameters can be used to obtain the estimated relative effect of any treatment compared to another. For example, the relative effect of treatment D against treatment C is D – C = (D – A) – (C – A) = $\delta^{AD}$- $\delta^{AC}$. With this in mind, the vector of basic parameter estimates, $\hat{\boldsymbol{\delta}}$, is used to create a distance matrix ***D****_1_* of all absolute relative treatment effect estimates, where the entries of ***D****_1_* are the absolute values of the matrix

$\left[\begin{matrix} \delta^{AA}-\delta^{AA} & \delta^{AB}-\delta^{AA} & \delta^{AC}-\delta^{AA} & \cdots& \delta^{At}-\delta^{AA} \\ \delta^{AA}-\delta^{AB} & \delta^{AB}-\delta^{AB} & \cdots& \cdots& \delta^{At}-\delta^{AB} \\ \delta^{AA}-\delta^{AC} & \vdots& \ddots& & \delta^{At}-\delta^{AC} \\ \vdots& \vdots& & \ddots& \vdots\\ \delta^{AA}-\delta^{At} & \delta^{AB}-\delta^{At} & \delta^{AC}-\delta^{At} & \cdots& \delta^{At}-\delta^{At} \end{matrix}\right]$.

Here, hats ($\hat{\delta}^{AB}$), to denote estimates, have been omitted for clarity; here we define the relative treatment effect of A to itself to be zero.

**Function arguments**

The function loads the packages mvtnorm, igraph and gplots.

The function requires two arguments with respect to the data:

- ests: a vector of treatment effect estimates relative to a single reference treatment, which hence has length t-1;
- cov: a square covariance matrix of dimension t-1.

The following arguments are used to choose either the first or second method, and whether the unweighted or weighted approach should be taken:

- method: Either “community” or “bootstrap”. If method=”community”, undertake community detection to group similar treatments with respect to relative treatment effect estimates, standard errors and p values, then plot. If method=”bootstrap”, use bootstrap replication to obtain *n* sets of relative treatment effect estimates and covariance matrices. Undertake community detection on each of the *n* replications, and visualise the proportion of times each pair of treatments is in the same community. Default is “community”;
- weighted: TRUE/FALSE. Detect communities using weighted or unweighted adjacency matrices. Default is FALSE.

Finally, there are the following miscellaneous optional arguments:

- quants: A scalar or vector denoting what quantile(s) of the estimated effects (and, if method=”community”, standard errors and p values) should be used as the threshold(s) to create the adjacency matrices. Default is quartiles, i.e., 20%, 40%, 60% and 80%;
- fix.layout: TRUE/FALSE. For method="community", fixes plotted location of each node when TRUE, otherwise R chooses a “best” layout, which changes from quantile to quantile. For method="bootstrap", returns heat maps with treatments ordered alphabetically when TRUE. Otherwise, the first heat map returned has treatment order chosen by R, and subsequent heat maps will have the same ordering. Default is FALSE if method=“community”, TRUE if method=“bootstrap”
- fast: TRUE/FALSE. If TRUE, R will seek to maximise modularity using the edge betweenness algorithm (Newman and Girvan, 2004). Otherwise, all possible sets of communities (and thus all modularities) will be examined. Default is FALSE;
- n: Number of bootstrap replications to create ("bootstrap" method only). Default is 1000;
- seed: Sets seed, for reproducible plots (and results when using "bootstrap" method); Default is 1.
- save: If TRUE, saves output plots as .pdf files. Default is FALSE.

Also contained in this document is R code that loads an example dataset in the correct format, then runs some examples.

**Function code**

cd <- function(ests, Cov, method="community", weighted=FALSE, quants=c(0.2, 0.4, 0.6, 0.8),

fix.layout=if(method=="community")FALSE else TRUE, fast=FALSE, n=1000, seed=1, save=FALSE)

#

# ests: Vector of treatment effect estimates

#

# Cov: covariance matrix

#

# method: "community": Undertake community detection to group similar treatments WRT relative treatment effect

# estimates, SEs and p values, then plot.

# "boostrap": Use bootstrap replication to obtain n sets of relative trt effect estimates and covariance matrices.

# Undertake community detection on each of the n replications, and visualise the proportion of times

# each pair of treatments is in the same community.

#

# weighted: FALSE: Detect communities using unweighted adjacency matrices (i.e. thresholds only)

# TRUE: Detect communities using weighted adjacency matrices (i.e. combining thresholds and weights)

#

# quants: A scalar or vector of the quantiles to be used as thresholds in the creation of the adjacency matrices that form

# the basis of both methods. Default is quartiles (20, 40, 60, 80 percentiles).

#

#

# fix.layout: For "community" method, fixes location of each node when plotting when TRUE. For "bootstrap" method,

# fix.layout=TRUE returns heatmaps with treatments ordered alphabetically. When fix.layout=FALSE, the

# first heatmap returned has treatments ordered by R, and subsequent heatmaps with the same ordering.

#

# fast: TRUE: Speeds up code by using the cluster edge betweenness algorithm to find the best set of communities.

# FALSE: Searches over all possible sets of communities.

#

# n: Number of bootstrap replications to create ("bootstrap" method only)

#

# seed: Sets seed, for reproduceable plots (and results when using "bootstrap" method)

#

# save: If TRUE, saves plots as pdfs.

#

{

require(mvtnorm)

require(igraph)

require(gplots)

set.seed(seed)

############## FUNCTION TO CREATE SQUARE MATRICES OF EFFECT ESTIMATES, SEs AND P VALUES #####################

fns <- function(est, covar=NULL)

{

# Create estimates matrix:

est <- c(0, est)

mat <- matrix(rep(est, each=length(est)), nrow=length(est)) - matrix(rep(est, times=length(est)), nrow=length(est))

if(is.null(covar)) return(mat)

else{

# Create SEs matrix

trts <- nrow(covar)+1

se_mat <- matrix(0, nrow=nrow(covar)+1, ncol=nrow(covar)+1)

# First row and column is simply square root of the diagonal of the covariance matrix:

se_mat[1, ] <- c(0, diag(covar))^0.5

se_mat[, 1] <- c(0, diag(covar))^0.5

# For remaining elements, use variance-covariance of linear combinations: var(AY)= A * Y * t(A)

for(i in 1:nrow(covar))

{

for(j in 1:ncol(covar))

{

lin_com <- rep(0, nrow(covar))

lin_com[i] <- lin_com[i] - 1

lin_com[j] <- lin_com[j] + 1

se_mat[i+1,j+1] <- (t(lin_com) %*% covar %*% lin_com)^0.5

}

}

# Create p value matrix

z <- mat/se_mat

p <- 2 * pnorm(-abs(z))

diag(p) <- 1

return(list(est=abs(mat), se=se_mat, p=1-p)) # We take absolute treatment effect estimates, and compliment of p value.

}

}

########################## END OF INITIAL FUNCTION #############################

############################### MAIN FUNCTION ##################################

# Create required square matrices using above function:

matrices <- fns(est=ests, covar=Cov)

est <- matrices$est

se <- matrices$se

p <- matrices$p

trts <- LETTERS[1:nrow(est)]

weights <- switch(weighted + 1, NULL, TRUE) # If weighted==FALSE, weights=NULL. If weighted==TRUE, weights=TRUE.

################### COMMUNITY DETECTION WITHOUT SIMULATION ###############

if(method=="community")

{

rownames(est) <- trts

colnames(est) <- trts

rownames(se) <- trts

colnames(se) <- trts

rownames(p) <- trts

colnames(p) <- trts

# Create vectors for finding quantiles. Take the lower triangle of each matrix:

est.vec <- est[c(lower.tri(est))]

se.vec <- se[c(lower.tri(se))]

p.vec <- p[c(lower.tri(p))]

# Find quantiles

est.quant <- quantile(est.vec, probs=quants)

se.quant <- quantile(se.vec, probs=quants)

p.quant <- quantile(p.vec, probs=quants)

# Create three empty lists, to be populated with the adjacency matrices:

est.adj.mat <- vector("list", length(quants))

se.adj.mat <- vector("list", length(quants))

p.adj.mat <- vector("list", length(quants))

names(est.adj.mat) <- paste("est", as.character(100*quants), sep="")

names(se.adj.mat) <- paste("se", as.character(100*quants), sep="")

names(p.adj.mat) <- paste("p", as.character(100*quants), sep="")

par(mfrow=c(1,3))

for(i in 1:length(quants))

{

################## UNWEIGHTED ADJACENCY MATRICES #########################

if(weighted==FALSE)

{

# Create unweighted adjacency matrices: If est/se/p is lower than threshold -> 1; otherwise -> 0

est.adj.mat[[i]] <- 1*(est<est.quant[i])

se.adj.mat[[i]] <- 1*(se<se.quant[i])

p.adj.mat[[i]] <- 1*(p<p.quant[i])

}

################## WEIGHTED ADJACENCY MATRICES #########################

if(weighted==TRUE)

{

# In weighted adjacency matrices, larger numbers -> more weight -> more similar,

# therefore take reciprocal of est, SE and (compliment of) p for non-zero adjacencies (i.e. > 0)

est.adj.mat[[i]] <- est

est.adj.mat[[i]][est.adj.mat[[i]]>=est.quant[i]] <- 0 # If estimated trt effect contrast is large, no connection

est.adj.mat[[i]][est.adj.mat[[i]] > 0] <- 1 / est.adj.mat[[i]][est.adj.mat[[i]] > 0]

se.adj.mat[[i]] <- se

se.adj.mat[[i]][se.adj.mat[[i]]>=se.quant[i]] <- 0 # If estimated SE is large, no connection

se.adj.mat[[i]][se.adj.mat[[i]] > 0] <- 1 / se.adj.mat[[i]][se.adj.mat[[i]] > 0]

p.adj.mat[[i]] <- p

p.adj.mat[[i]][p.adj.mat[[i]]>=p.quant[i]] <- 0 # If this p is large (ie original p value is small), no connection

p.adj.mat[[i]][p.adj.mat[[i]] > 0] <- 1 / p.adj.mat[[i]][p.adj.mat[[i]] > 0]

}

# Graphs:

g.est <- graph_from_adjacency_matrix(est.adj.mat[[i]], mode="undirected", diag=F, weighted=weights)

g.se <- graph_from_adjacency_matrix(se.adj.mat[[i]], mode="undirected", diag=F, weighted=weights)

g.p <- graph_from_adjacency_matrix(p.adj.mat[[i]], mode="undirected", diag=F, weighted=weights)

if(fast)

{

ceb.est <- cluster_edge_betweenness(g.est)

ceb.se <- cluster_edge_betweenness(g.se)

ceb.p <- cluster_edge_betweenness(g.p)

}

else

{

ceb.est <- cluster_optimal(g.est)

ceb.se <- cluster_optimal(g.se)

ceb.p <- cluster_optimal(g.p)

}

########## PLOTTING ############

if(fix.layout==FALSE)

{

layout.est <- layout_nicely(g.est)

layout.se <- layout_nicely(g.se)

layout.p <- layout_nicely(g.p)

}

else

{

browser()

layout.est <- layout_in_circle(g.est)

layout.se <- layout_in_circle(g.se)

layout.p <- layout_in_circle(g.p)

}

plot(ceb.est, g.est, layout=layout.est)

title(paste("Est: ", names(est.quant[i]), " (", round(est.quant[i],2), ")", sep=""), cex.main=2)

plot(ceb.se, g.se, layout=layout.se)

title(paste("SE: ", names(se.quant[i]), " (", round(se.quant[i],2), ")", sep=""),cex.main=2)

plot(ceb.p, g.p, layout=layout.p)

title(paste("P: ", names(p.quant[i]), " (", round(1-p.quant[i],2), ")", sep=""), cex.main=2)

if(i!=length(quants))

{

cat("Press [enter] to continue")

line <- readline()

}

if(save==TRUE) dev.print(pdf, paste(100*quants[i], " percentile",".pdf", sep=""))

} # end of for loop i in 1:length(quants)

} # end of if statement <if method=="community">

####################### HEATMAP/BOOTSTRAPPING METHOD #######################

if(method=="bootstrap")

{

# Create bootstrap replications (p-1 columns, n rows):

pred <- rmvnorm(n, mean=ests, sigma=Cov)

# Create lists of length n for storing

# - the n square matrices of relative treatment effect estimates

# - the n vectors containing the lower triangle of those matrices

# - the n adjacency matrices:

rel.effects.list <- vector("list", n)

est.vec.pred <- vector("list", n)

est.adj.mat.list <- vector("list", n)

# Create a list of matrices, each matrix to hold n rows of treatment memberships:

membership.list <- vector("list", length(quants))

# All possible pairs of treatments:

pair <- combn(trts, 2)

pair <- t(pair)

for(i in 1:n)

{

# Create matrix of relative effects:

rel.effects.list[[i]] <- fns(pred[i,])

rel.effects.list[[i]] <- abs(rel.effects.list[[i]])

# Make vector out of lower triangle of matrix:

est.vec.pred[[i]] <- c(rel.effects.list[[i]])[c(lower.tri(rel.effects.list[[i]]))]

}

# Find the quantiles for each bootstrap replication:

est.quant.pred <- lapply(est.vec.pred, quantile, probs=quants)

for(j in 1:length(quants))

{

if(weighted==TRUE) est.adj.mat.list <- rel.effects.list

for(i in 1:n)

{

if(weighted==TRUE)

{

est.adj.mat.list[[i]][est.adj.mat.list[[i]]>=est.quant.pred[[i]][j]] <- 0 # If estimated trt effect contrast is large, no connection

est.adj.mat.list[[i]][est.adj.mat.list[[i]] > 0] <- 1 / est.adj.mat.list[[i]][est.adj.mat.list[[i]] > 0] # Take reciprocal of non-zero elements.

}

else{

est.adj.mat.list[[i]] <- 1*(rel.effects.list[[i]]<est.quant.pred[[i]][j]) # If trt contrast is below threshold, -> 1

}

} # end of 1:n

# Create and store all n memberships for current quantile:

ceb.list <- if(fast) lapply(est.adj.mat.list, function(x) cluster_edge_betweenness(graph_from_adjacency_matrix(x, mode="undirected", diag=F, weighted = weights))$membership)

else lapply(est.adj.mat.list, function(x) cluster_optimal(graph_from_adjacency_matrix(x, mode="undirected", diag=F, weighted = weights))$membership)

# Collapse n memberships into a matrix of n rows

membership.list[[j]] <- matrix(data=unlist(ceb.list), byrow=TRUE, nrow=n, dimnames=list(1:n, LETTERS[1:(ncol(pred)+1)]))

# For the current quantile, there now exists n sets of detected communities. How often does each unique pair of trts appear in the same community?

n.same.comm <- apply(pair, 1, function(x) sum(membership.list[[j]][,x[1]]==membership.list[[j]][,x[2]]))

prob.same.comm <- n.same.comm/nrow(pred)

# Create symmetric matrix of these proportions:

mat <- matrix(NA, nrow=ncol(pred)+1, ncol=ncol(pred)+1, dimnames=list(trts, trts))

mat[c(lower.tri(mat))] <- prob.same.comm

mat[upper.tri(mat)] <- t(mat)[upper.tri(mat)]

if(mean(mat, na.rm=T)!=1) # Make sure at least some probabilities are != 1 (requirement of heatmap.2 function)

{

fix.rows <- ifelse(j==1 & fix.layout==FALSE, yes=TRUE, no=FALSE) # For reordered layout (used in call to heatmap.2 below).

if(j>1 & fix.layout==FALSE) mat <- mat[row.order, row.order] # For reordered layout, use row order from first heatmap (ie when j==1)

heat <- heatmap.2(mat, dendrogram = "none", trace="none", cellnote=round(mat,2), notecol=1,

density.info = "none", col=rev(heat.colors(16)), symm=TRUE,

breaks=seq(from=0, to=1, length=17),

notecex=4*(1/sqrt(ncol(mat))),

srtCol=0,

main=paste("Proportion of times\ntreatments in same community (", names(est.quant.pred[[1]][j]), " quantile)" ,sep=""),

Rowv=fix.rows, Colv=fix.rows, revC=fix.rows

)

if(j==1) row.order <- heat$rowInd # Recording row order of first heatmap.

if(j!=length(quants))

{

cat("Press [enter] to continue")

line <- readline()

}

filetext <- ifelse(weighted, "weighted", "unweighted")

if(save==TRUE) dev.print(pdf, paste("Predictive ests (", filetext, "), ", 100*quants[j], ".pdf", sep=""))

}

# If all probabilities = 1, don't attempt heatplot:

else print(paste("For", names(est.quant.pred[[1]][j]), " quantile, all treatments in the same community for all simulations; no heatmap produced."),q=F)

}

} # end of method=="bootstrap" if statement

}

**Example dataset**

oak <- structure(list(est = structure(c(0.0408427031130733, 0.598289533357103,

-0.776237460184288, -0.458010905158822, -0.14916168, -0.593975186396551,

-0.372743568115302, -0.0288447116630532, -0.00874969404766398,

-0.283846436000015, -0.347788489848219, -0.254615262827929, -0.253284937355232,

-1.111864761692, -0.246939725936896, -0.288379274336666, 0.45557099,

-0.704507195305565, 0.00710261092398497, -0.781897596886927,

-0.62780514834371), .Dim = c(21L, 1L), .Dimnames = list(NULL,

NULL)), Cov = structure(c(0.0413184069819484, 0.0189377322676937,

0.00509210209786147, 0.0353248067308859, 0, 0.000958358798725847,

0.00661182735122684, 0.0274885620750255, 0.0413184069819484,

0, 0.000195323036789215, 0.00458355905922423, 0.0286273473224269,

0.0352885460740222, 0.0413184069819484, 0.0135679010297421, 0,

0.0311314875186361, 0.0413184069819484, 0.0413184069819484, 0.0269976957102873,

0.0189377322676937, 0.101880523246978, 0.00377967469956747, 0.0161906467635552,

0, 0.00138729561512657, 0.00957111168022458, 0.0175223261498405,

0.0189377322676937, 0, 0.000282744618018997, 0.00340220244550847,

0.0319824158771902, 0.041284399618242, 0.0189377322676937, 0.00701877020643887,

0, 0.016188483837477, 0.0189377322676937, 0.0189377322676937,

0.0185881264791009, 0.00509210209786147, 0.00377967469956747,

0.02560880537809, 0.0043534476665444, 0, 0.000256341031385051,

0.00176852619791956, 0.00472649986865891, 0.00509210209786147,

0, 5.22448468886345e-05, 0.0230512801257363, 0.00720018191488496,

0.0047385044175188, 0.00509210209786147, 0.00178877704411337,

0, 0.00411658101848248, 0.00509210209786147, 0.00509210209786147,

0.00639187860540716, 0.0353248067308859, 0.0161906467635552,

0.0043534476665444, 0.0642392952529158, 0, 0.000819340381603407,

0.00565272333519813, 0.0235011031048334, 0.0353248067308859,

0, 0.000166989703346578, 0.00391867329196489, 0.0244746974834843,

0.0301696304609083, 0.0353248067308859, 0.011599757024244, 0,

0.0266155900037787, 0.0353248067308859, 0.0353248067308859, 0.0230814412463148,

0, 0, 0, 0, 0.212570273797084, 0, 0, 0, 0, 0, 0, 0, 0, 0, 0,

0, 0, 0, 0, 0, 0, 0.000958358798725847, 0.00138729561512657,

0.000256341031385051, 0.000819340381603407, 0, 0.0476890970211617,

0.00179109813988969, 0.00114221885883785, 0.000958358798725847,

0, 0.00971951138163803, 0.00023074051424642, 0.000957440464467023,

0.00107392409665396, 0.000958358798725847, 0.000515041701007569,

0, 0.00120278071216784, 0.000958358798725847, 0.000958358798725847,

0.000817434111774369, 0.00661182735122684, 0.00957111168022458,

0.00176852619791956, 0.00565272333519813, 0, 0.00179109813988969,

0.0123569916463426, 0.0078802990090892, 0.00661182735122684,

0, 0.000365043581105417, 0.00159190529179568, 0.00660549165777035,

0.00740912560602417, 0.00661182735122684, 0.00355333181087473,

0, 0.00829812218640086, 0.00661182735122684, 0.00661182735122684,

0.00563957176084914, 0.0274885620750255, 0.0175223261498405,

0.00472649986865891, 0.0235011031048334, 0, 0.00114221885883785,

0.0078802990090892, 0.0922787930706752, 0.0274885620750255, 0,

0.000232795542215231, 0.00425446915145554, 0.0319862647449734,

0.024803432284628, 0.0274885620750255, 0.00945242581655177, 0,

0.0217332327169195, 0.0274885620750255, 0.0274885620750255, 0.0269774119643,

0.0413184069819484, 0.0189377322676937, 0.00509210209786147,

0.0353248067308859, 0, 0.000958358798725847, 0.00661182735122684,

0.0274885620750255, 0.123538079891138, 0, 0.000195323036789215,

0.00458355905922423, 0.0286273473224269, 0.0352885460740222,

0.0413184069819484, 0.0135679010297421, 0, 0.0311314875186361,

0.0413184069819484, 0.0413184069819484, 0.0269976957102873, 0,

0, 0, 0, 0, 0, 0, 0, 0, 0.0822205984320331, 0, 0, 0, 0, 0, 0,

0, 0, 0, 0, 0, 0.000195323036789215, 0.000282744618018997, 5.22448468886345e-05,

0.000166989703346578, 0, 0.00971951138163803, 0.000365043581105417,

0.000232795542215231, 0.000195323036789215, 0, 0.0665419380842615,

4.70272073599531e-05, 0.000195135871150981, 0.000218876391721397,

0.000195323036789215, 0.000104970611474147, 0, 0.000245138649120206,

0.000195323036789215, 0.000195323036789215, 0.000166601186631917,

0.00458355905922423, 0.00340220244550847, 0.0230512801257363,

0.00391867329196489, 0, 0.00023074051424642, 0.00159190529179568,

0.00425446915145554, 0.00458355905922423, 0, 4.70272073599531e-05,

0.0539613504748291, 0.00648110709679082, 0.00426527481827471,

0.00458355905922423, 0.00161013370665162, 0, 0.00370546227425804,

0.00458355905922423, 0.00458355905922423, 0.00575352821373703,

0.0286273473224269, 0.0319824158771902, 0.00720018191488496,

0.0244746974834843, 0, 0.000957440464467023, 0.00660549165777035,

0.0319862647449734, 0.0286273473224269, 0, 0.000195135871150981,

0.00648110709679082, 0.135107145714988, 0.0295312788160067, 0.0286273473224269,

0.00964813724121964, 0, 0.0221635910988096, 0.0286273473224269,

0.0286273473224269, 0.0280974401012796, 0.0352885460740222, 0.041284399618242,

0.0047385044175188, 0.0301696304609083, 0, 0.00107392409665396,

0.00740912560602417, 0.024803432284628, 0.0352885460740222, 0,

0.000218876391721397, 0.00426527481827471, 0.0295312788160067,

0.230878749404197, 0.0352885460740222, 0.0118034167844491, 0,

0.0271055037225967, 0.0352885460740222, 0.0352885460740222, 0.0247319672008902,

0.0413184069819484, 0.0189377322676937, 0.00509210209786147,

0.0353248067308859, 0, 0.000958358798725847, 0.00661182735122684,

0.0274885620750255, 0.0413184069819484, 0, 0.000195323036789215,

0.00458355905922423, 0.0286273473224269, 0.0352885460740222,

0.131657315268973, 0.0135679010297421, 0, 0.0311314875186361,

0.0413184069819484, 0.0413184069819484, 0.0269976957102873, 0.0135679010297421,

0.00701877020643887, 0.00178877704411337, 0.011599757024244,

0, 0.000515041701007569, 0.00355333181087473, 0.00945242581655177,

0.0135679010297421, 0, 0.000104970611474147, 0.00161013370665162,

0.00964813724121964, 0.0118034167844491, 0.0135679010297421,

0.0912110296076178, 0, 0.0106284696257306, 0.0135679010297421,

0.0135679010297421, 0.0090267432039417, 0, 0, 0, 0, 0, 0, 0,

0, 0, 0, 0, 0, 0, 0, 0, 0, 0.299372353797084, 0, 0, 0, 0, 0.0311314875186361,

0.016188483837477, 0.00411658101848248, 0.0266155900037787, 0,

0.00120278071216784, 0.00829812218640086, 0.0217332327169195,

0.0311314875186361, 0, 0.000245138649120206, 0.00370546227425804,

0.0221635910988096, 0.0271055037225967, 0.0311314875186361, 0.0106284696257306,

0, 0.104408426938182, 0.0311314875186361, 0.0311314875186361,

0.020728755040381, 0.0413184069819484, 0.0189377322676937, 0.00509210209786147,

0.0353248067308859, 0, 0.000958358798725847, 0.00661182735122684,

0.0274885620750255, 0.0413184069819484, 0, 0.000195323036789215,

0.00458355905922423, 0.0286273473224269, 0.0352885460740222,

0.0413184069819484, 0.0135679010297421, 0, 0.0311314875186361,

0.0999968771143836, 0.0413184069819484, 0.0269976957102873, 0.0413184069819484,

0.0189377322676937, 0.00509210209786147, 0.0353248067308859,

0, 0.000958358798725847, 0.00661182735122685, 0.0274885620750255,

0.0413184069819484, 0, 0.000195323036789215, 0.00458355905922423,

0.0286273473224269, 0.0352885460740222, 0.0413184069819484, 0.0135679010297421,

0, 0.0311314875186361, 0.0413184069819484, 0.323009570779033,

0.0269976957102873, 0.0269976957102873, 0.0185881264791009, 0.00639187860540716,

0.0230814412463148, 0, 0.000817434111774369, 0.00563957176084914,

0.0269774119643, 0.0269976957102873, 0, 0.000166601186631917,

0.00575352821373703, 0.0280974401012796, 0.0247319672008902,

0.0269976957102873, 0.0090267432039417, 0, 0.020728755040381,

0.0269976957102873, 0.0269976957102873, 0.0489889776452823), .Dim = c(21L,

21L), .Dimnames = list(NULL, NULL))), .Names = c("est", "Cov"

))

**Example code**

cd(ests=oak$est, Cov=oak$Cov, method = "community", weighted=FALSE, seed=1)

cd(ests=oak$est, Cov=oak$Cov, method = "community", weighted=TRUE, seed=1)

cd(ests=oak$est, Cov=oak$Cov, method = "bootstrap", weighted=FALSE, seed=1)

cd(ests=oak$est, Cov=oak$Cov, method = "bootstrap", weighted=TRUE, seed=1)

**Example code output**

**First method, unweighted approach**

**
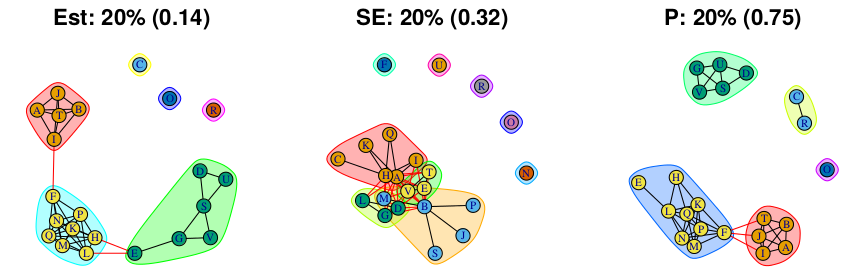
**

Figure S1: Groupings for example dataset, using method=“community” with unweighted approach and threshold at 20% quantile.


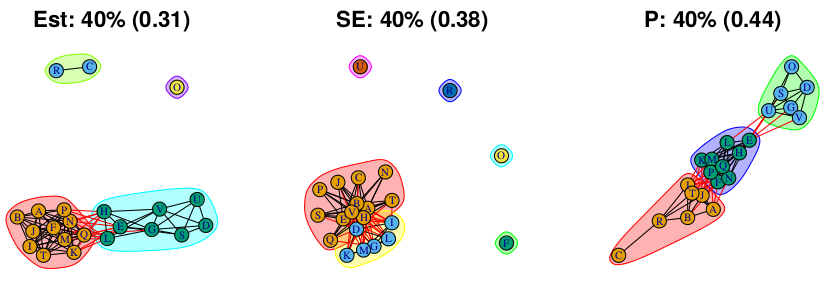


Figure S2: Groupings for example dataset, using method=“community” with unweighted approach and threshold at 40% quantile.


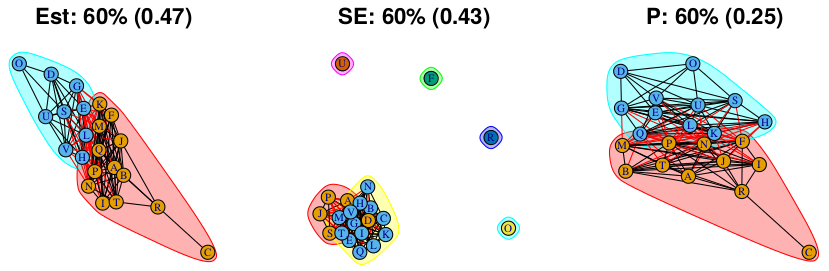


Figure S3: Groupings for example dataset, using method=“community” with unweighted approach and threshold at 60% quantile.


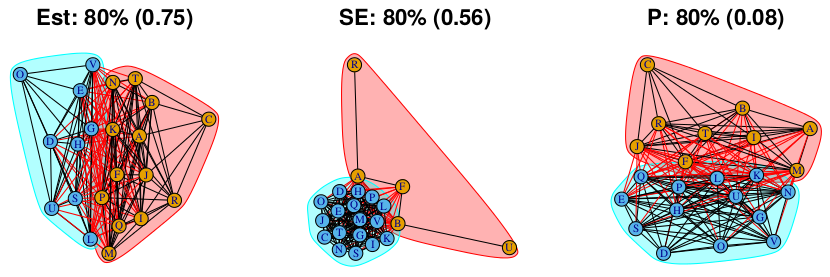


Figure S4: Groupings for example dataset, using method=“community” with unweighted approach and threshold at 80% quantile.

**First method, weighted approach**

**
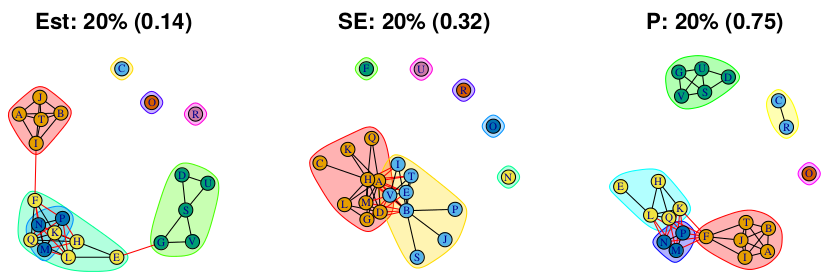
**

Figure S5: Groupings for example dataset, using method=“community” with weighted approach and threshold at 20% quantile.


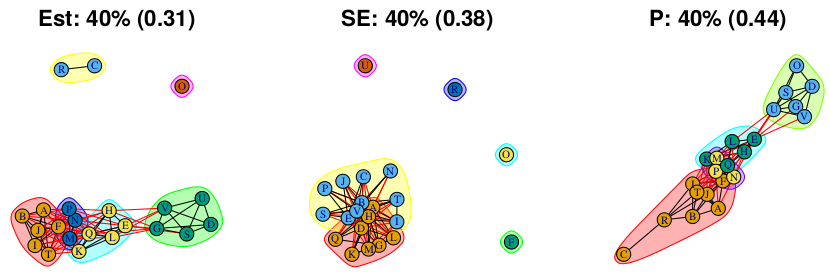


Figure S6: Groupings for example dataset, using method=“community” with weighted approach and threshold at 40% quantile.


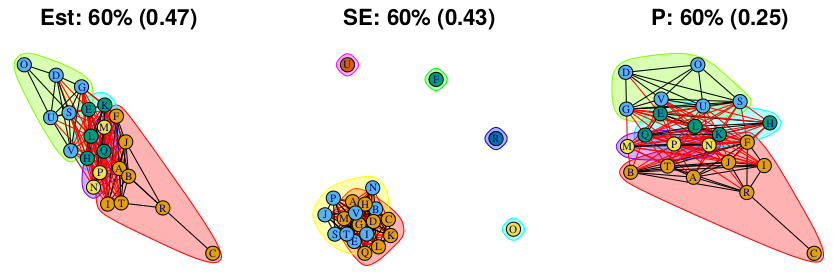


Figure S7: Groupings for example dataset, using method=“community” with weighted approach and threshold at 60% quantile.


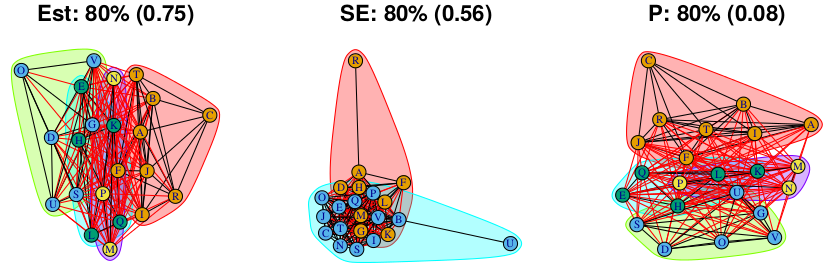


Figure S8: Groupings for example dataset, using method=“community” with weighted approach and threshold at 80% quantile.

**Second method, unweighted approach**

**
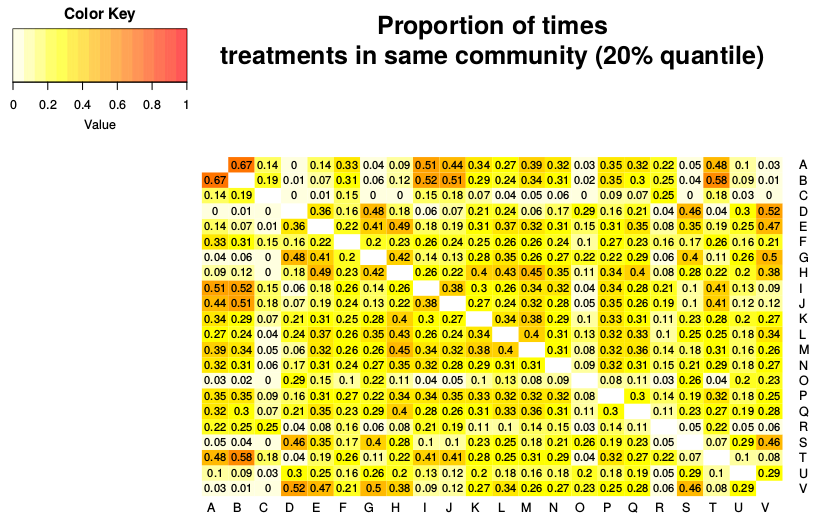
**

Figure S9: Proportion of times treatments are grouped together for example dataset, using method=“bootstrap” with unweighted approach and threshold at 20% quantile.

**
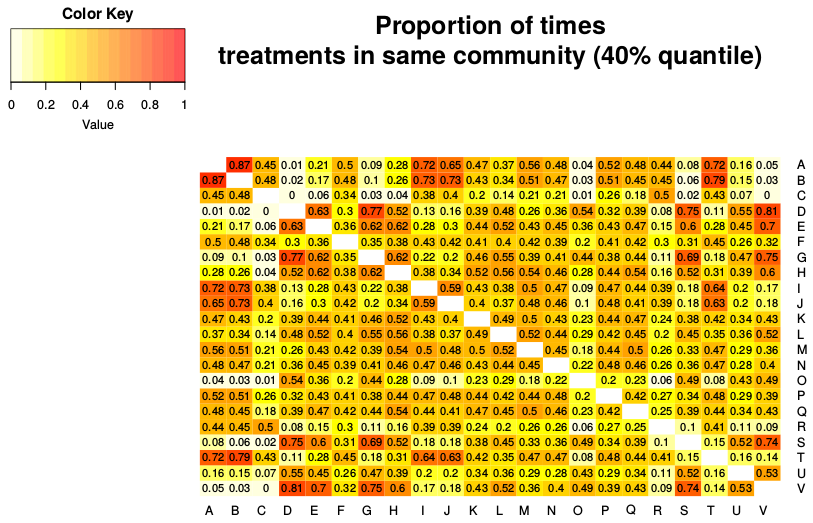
**

Figure S10: Proportion of times treatments are grouped together for example dataset, using method=“bootstrap” with unweighted approach and threshold at 40% quantile.


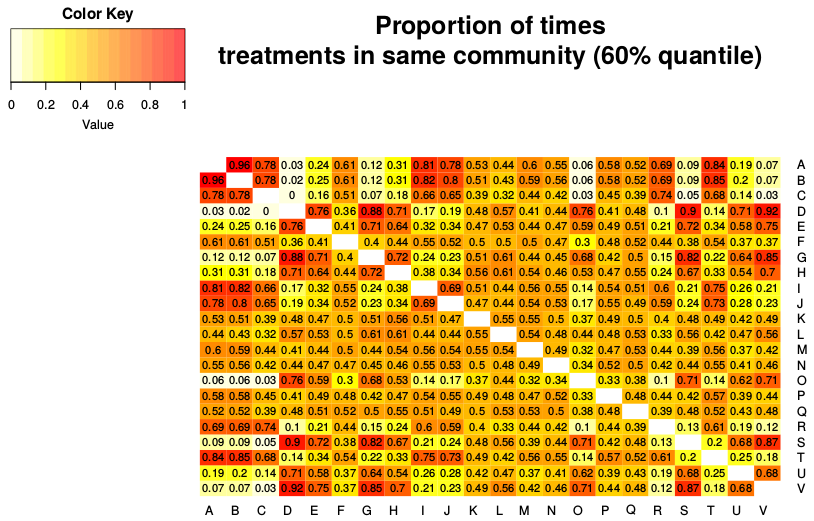


Figure S11: Proportion of times treatments are grouped together for example dataset, using method=“bootstrap” with unweighted approach and threshold at 60% quantile.


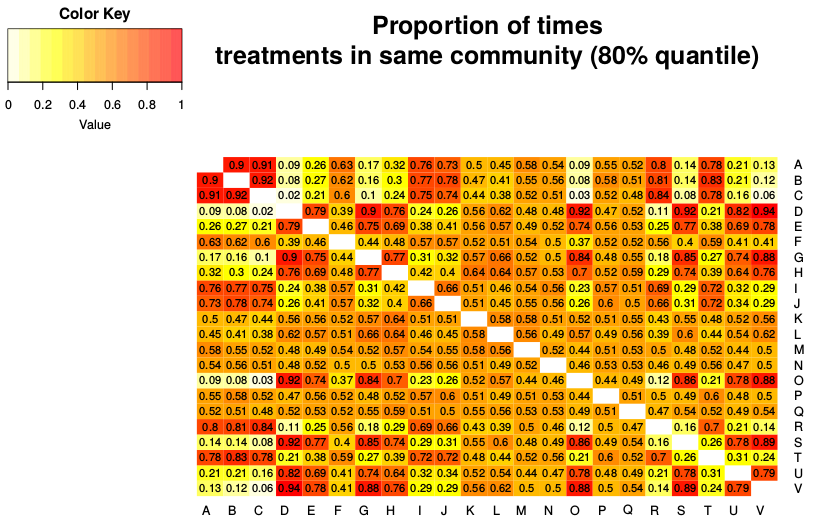


Figure S12: Proportion of times treatments are grouped together for example dataset, using method=“bootstrap” with unweighted approach and threshold at 80% quantile.

**Second method, weighted approach**

**
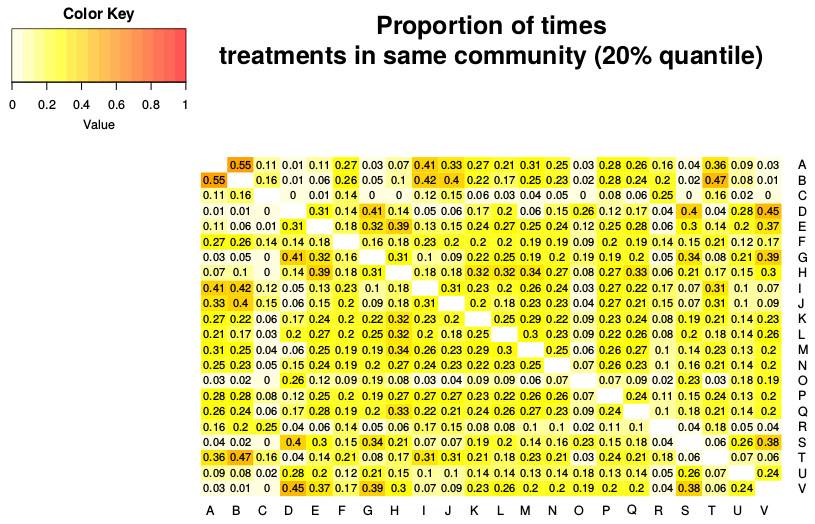
**

Figure S13: Proportion of times treatments are grouped together for example dataset, using method=“bootstrap” with weighted approach and threshold at 20% quantile.


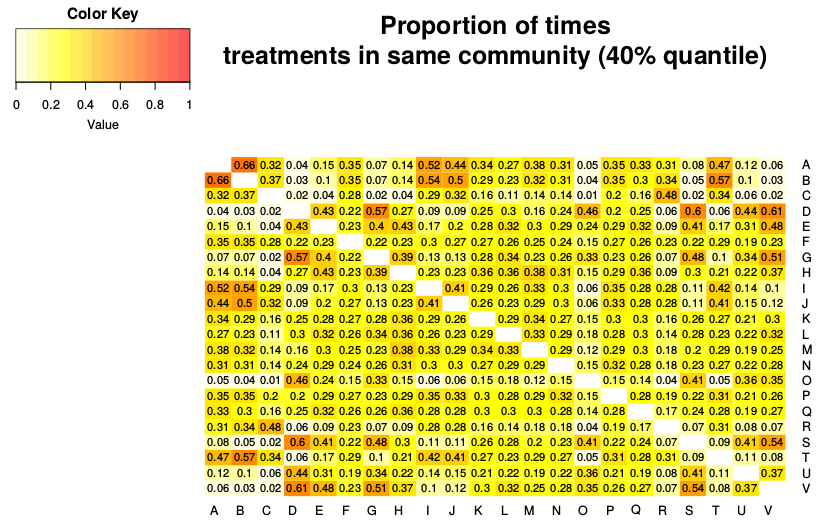


Figure S14: Proportion of times treatments are grouped together for example dataset, using method=“bootstrap” with weighted approach and threshold at 40% quantile.

**
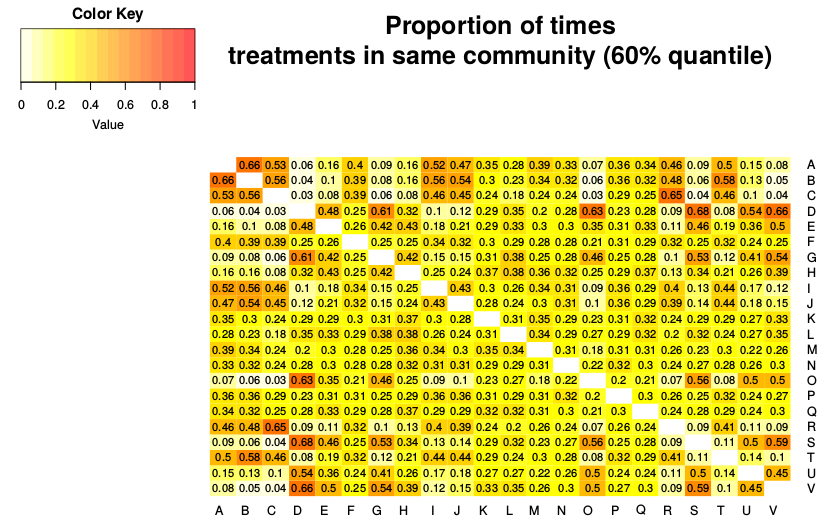
**

Figure S15: Proportion of times treatments are grouped together for example dataset, using method=“bootstrap” with weighted approach and threshold at 60% quantile.

**
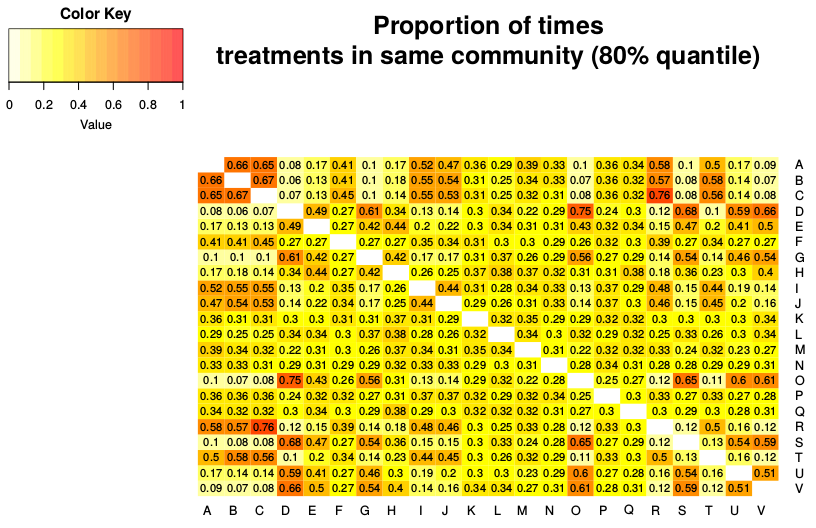
**

Figure S16: Proportion of times treatments are grouped together for example dataset, using method=“bootstrap” with weighted approach and threshold at 80% quantile.

**References**

M Newman and M Girvan: Finding and evaluating community structure in networks, Physical Review E 69, 026113 (2004)
